# Unveiling CXCR2 as a promising therapeutic target in renal cell carcinoma: exploring the immunotherapeutic paradigm shift through its inhibition by RCT001

**DOI:** 10.1186/s13046-024-02984-2

**Published:** 2024-03-19

**Authors:** Christopher Montemagno, Arnaud Jacquel, Charlotte Pandiani, Olivia Rastoin, Rosie Dawaliby, Thomas Schmitt, Maxence Bourgoin, Héliciane Palenzuela, Anne-Laure Rossi, Damien Ambrosetti, Jerome Durivault, Frederic Luciano, Delphine Borchiellini, Julie Le Du, Leticia Christina Pires Gonçalves, Patrick Auberger, Rachid Benhida, Lisa Kinget, Benoit Beuselinck, Cyril Ronco, Gilles Pagès, Maeva Dufies

**Affiliations:** 1 Biomedical Department, Centre Scientifique de Monaco (CSM), 98000 Monaco, Monaco; 2 INSERM U1065/C3M, 06204 Nice, France; 3 Institute for Research On Cancer and Aging (IRCAN), UMR 7284 and INSERM U1081, Université Côte d’Azur, CNRS, 33 Avenue de Valombrose, 06107 Nice, France; 4 Roca Therapeutics, 06000 Nice, France; 5 G.CLIPS Biotech, Labège, France; 6 Department of Pathology, Université Côte d’Azur, CHU Nice, Nice, France; 7 Centre Antoine Lacassagne, Department of Medical Oncology, Université Côte d’Azur, Nice, France; 8 Institut de Chimie de Nice, UMR 7272, Université Côte d’Azur, CNRS, 06108 Nice, France; 9 Department of General Medical Oncology, University Hospitals Leuven, 3000 Louvain, Belgium; 10 Laboratory of Experimental Oncology, Department of Oncology, KU Leuven, 3000 Louvain, Belgium; 11 Institut Universitaire de France (IUF), Paris, France

**Keywords:** Renal Cell Carcinoma, M2 tumor associated macrophages, Immunotherapies resistance, CXCR2 inhibitors, Nivolumab, Ipilimumab

## Abstract

**Background:**

In clear cell renal cell carcinoma (ccRCC), first-line treatment combines nivolumab (anti-PD-1) and ipilimumab (anti-CTLA4), yielding long-term remissions but with only a 40% success rate. Our study explored the potential of enhancing ccRCC treatment by concurrently using CXCR2 inhibitors alongside immunotherapies.

**Methods:**

We analyzed ELR + CXCL levels and their correlation with patient survival during immunotherapy. RCT001, a unique CXCR2 inhibitor, was examined for its mechanism of action, particularly its effects on human primary macrophages. We tested the synergistic impact of RCT001 in combination with immunotherapies in both mouse models of ccRCC and human ccRCC in the presence of human PBMC.

**Resuts:**

Elevated ELR + CXCL cytokine levels were found to correlate with reduced overall survival during immunotherapy. RCT001, our optimized compound, acted as an inverse agonist, effectively inhibiting angiogenesis and reducing viability of primary ccRCC cells. It redirected M2-like macrophages without affecting M1-like macrophage polarization directed against the tumor. In mouse models, RCT001 enhanced the efficacy of anti-CTLA4 + anti-PD1 by inhibiting tumor-associated M2 macrophages and tumor-associated neutrophils. It also impacted the activation of CD4 T lymphocytes, reducing immune-tolerant lymphocytes while increasing activated natural killer and dendritic cells. Similar effectiveness was observed in human RCC tumors when RCT001 was combined with anti-PD-1 treatment.

**Conclusions:**

RCT001, by inhibiting CXCR2 through its unique mechanism, effectively suppresses ccRCC cell proliferation, angiogenesis, and M2 macrophage polarization. This optimization potentiates the efficacy of immunotherapy and holds promise for significantly improving the survival prospects of metastatic ccRCC patients.

**Supplementary Information:**

The online version contains supplementary material available at 10.1186/s13046-024-02984-2.

## Background

Renal cell carcinoma (RCC) is a globally prevalent urological cancer, constituting approximately 4% of all cancer cases [1], with around 400,000 new diagnoses annually [2]. Clear cell RCC (ccRCC) is the most common subtype, accounting for 75% of RCC cases and a significant portion of cancer-related deaths [3].

While localized ccRCC can often be treated surgically, metastatic ccRCC (mccRCC) presents resistance to conventional therapies [4]. Over the past two decades, the development of tyrosine kinase inhibitors targeting vascular endothelial growth factor (VEGF) receptors (such as axitinib, sunitinib, cabozantinib) has substantially improved both progression-free survival (PFS) and overall survival (OS) in mccRCC patients [5–7].

Immunotherapy, particularly immune checkpoint inhibitors (ICI), has shown promise in the treatment of solid tumors, including ccRCC. First-line treatments often involve combinations of ICI, such as nivolumab (anti-PD-1) + ipilimumab (anti-CTLA-4, CHECKMATE 214)) [8] or ICI + TKI (tyrosine kinase inhibitors, *i.e*., anti-angiogenic agents), including pembrolizumab + axitinib (KEYNOTE-426) [9], nivolumab + cabozantinib (CHECKMATE 9ER, COSMIC-313) [10], avelumab + axitinib (JAVELIN Renal 101) [11], atezolizumab + bevacizumab (IMMotion151) [12], pembrolizumab + lenvatinib (CLEAR) [13]. These combinations have shown varying PFS, with ICI combinations lasting approximately 12 months and ICI + TKI combinations lasting approximately 16 to 23 months. However, in contrast to ICI + TKI, ICI combinations appear to show a better OS (56 months compared to 38–46 months) and a higher percentage of long-term responders (50 months compared to 24 months median duration of response).

Despite this progress, there is a need for further improvement in treatments, particularly in increasing the percentage of patients with long-term responses. ccRCC display a heightened inflammatory profile, marked by chronic inflammation driven by cytokines, fostering pathological angiogenesis, immune tolerance, cancer-associated fibroblast proliferation, and tumor-associated macrophage (TAM) polarization [14].

TAMs can be categorized into two distinct groups. M1-like TAMs possess anti-tumor properties, actively promote inflammation, and exhibit the ability to phagocytose cancer cells while presenting tumor antigens to the immune system. This activity helps to initiate an anti-tumor immune response and is often associated with a more favorable prognosis. In contrast, M2-like TAMs have tumor-friendly properties. They suppress inflammation, promote tissue repair, and favor tumor growth and metastasis. They also contribute to processes such as angiogenesis, tissue remodeling and suppression of cytotoxic immune cells, creating a microenvironment that is more immunotolerant and conducive to tumor growth. The secretion of immunosuppressive cytokines, such as IL-6, IL-10, and TGFβ, by M2-like TAMs leads to the polarization of effector T lymphocytes into regulatory T cells (Tregs), thus undermining the anti-tumor immune response. Accumulation of M2-like TAMs has been linked to resistance to immunotherapy in preclinical models across various cancer types, including ccRCC.

The pro-angiogenic and pro-inflammatory ELR + CXCL cytokines, which comprise CXCL1-3 and 5–8, bind to the G-protein-coupled CXCR2 receptor. Following activation of the receptor, signaling pathways such as PI3K/AKT or ERK are activated, which initiate several crucial processes: (i) stimulation of cell survival, proliferation and stemness (ii) shaping of the tumor microenvironment and (iii) promotion of the metastatic process [15]. These cytokines are induced following anti-angiogenic treatments [16]. In addition, increased expression of ELR + CXCL/CXCR2 has been associated with shorter survival in RCC patients [17] and elevated plasmatic CXCL5 and CXCL7 levels correlate with lower sunitinib efficacy in mccRCC [18, 19]. Considerable progress has been made in the development of small inhibitors targeting CXCR2, and their potential to effectively inhibit tumor growth in experimental ccRCC has been validated [17]. However, the combined effect of anti-CXCR2 agents with ICI has not yet been extensively studied in ccRCC.

This study aims to investigate the impact of CXCR2 inhibition on M2 macrophage polarization and assess its synergistic effects with ICIs in ccRCC. The findings may provide insights into a novel therapeutic approach to enhance immunotherapy efficacy in ccRCC.

## Methods

### RCT001

RCT001 (1-(3,5-dichlorophenyl)-3-(6-nitrobenzo[d]thiazol-2-yl) urea) has been synthesized according to the previously described procedure (Grytsai et al., in revision). Its purity has been assessed by ^1^H NMR, ^13^C NMR and HPLC (See Supporting information).

### Cell lines

The RCC cell lines (A498, 786-O, RENCA, all from ATCC®) were maintained in DMEM medium supplemented with 7.5% FBS. Endothelial cells (TIME) were cultured in vascular cell basal medium (ATCC®, PCS-100-030TM). Primary RCC cells were obtained from tumor fragments and subsequently cultured in a medium for kidney cells (PromoCell).

### Evaluation of CXCR2 activity

The validation assay utilized is a proprietary fluorescent-based method developed by G. CLIPS biotech (https://gclips-biotech.com/). This assay is designed to evaluate the conformational changes that occur in a receptor upon activation or inactivation [20]. See Supplementary methods for more information.

### Immunoblot analysis

Cellular lysis was performed using Laemmli buffer [17]. For protein analysis, the membranes were subjected to immunoblotting using the following antibodies: pERK1/2 (CST, 9102S), pAKT Ser473 (CST, 4051S), HSP90 (Abcam, ab1429).

### Matrigel-based tube formation assay

Matrigel (Corning) was dissolved at 4°C and 100 μL of the Matrigel solution was added to each well of the 48-well plate. The plate was then placed in a 37°C incubator for 30 min to allow the Matrigel to solidify. Then 20,000 cells were added to each well and cultured for 24 h in their respective growth medium. The TIME cells were cultured for 6 h before being photographed. This test allows the observation of tube formation of cells and provides information on the angiogenic potential and behavior of the tested cells.

### Migration assay of endothelial cells

The confluent TIME cells were starved for 2 h. The cell monolayer was then ruptured to create a scratch wound and rinsed with PBS. The cells were then treated with CXCL8 (75 ng/mL). Microscopic images were taken immediately after creating the scratch wound (0 h) using phase contract microscopy (EvosTM xl core, Thermo Fischer). Further images were taken after 4 and 8 h. To quantify the migration, the images were analyzed using the Java-based software ImageJ34. The software made it possible to measure the migration distance at the 4 h and 8 h time points.

### Migration assay of RCC cells

CXCL8-stimulated chemotaxis assays were monitored using modified Boyden chambers containing polycarbonate membranes (8-μm pores, Transwell; Corning, Sigma). Cells were seeded onto the upper side of the filters and chambers were placed on 24-well plates containing CXCL8 (75ng/ml) in presence/absence of RCT001. Cell migration was followed for 24 h at 37°C. Migratory cells on the lower membrane surface were fixed in 3% paraformaldehyde, stained with 0.1% crystal violet.

### Cell proliferation assay

50,000 TIME cells were seeded into individual wells of 6-well plates. The culture medium employed was Endothelial Cell Growth Medium sourced from Promocell, supplemented with 0.5% FBS. After an incubation period of 24 h, the cells were exposed to either CXCL7 or CXCL8 at a concentration of 75 ng/mL, marking the starting point (day 0) of the experiment. Following the 72-h mark, cell counting was performed. The outcome of the experiment was expressed as a percentage relative to day 0, allowing for the assessment of changes in cell proliferation over the experimental duration.

### Cell viability assay using XTT

10,000 cells were seeded per well in a 96-well plate, with a volume of 100 μl per well as already described [17].

### Colony formation assays

A total of 10,000 primary RCC cells were seeded for each experimental condition. After 10-day culture period, the colonies were identified and visualized. using GIEMSA staining. Images of the colonies were captured, and subsequently, the number of distinct colonies was quantified.

### PDX Tumorspheres establishment and treatment

Tumorspheres derived from MEXF486 (Charles River) patient-derived xenografts (PDXs) were cultured in a special procedure. The human tumor cells were coated with layers of Cypre's VersaGel® to promote the formation of 3D tumor spheres. These tumorspheres were cultured for 7 days together with fibroblasts (HDF) and pre-activated peripheral blood mononuclear cells (PBMCs). They were then treated with RCT001 and/or anti-PD-1 (pembrolizumab) at a concentration of 100 μg/mL at different doses for a further 7 days. The assessment process included quantification of tumorspheres, measurement of total tumor sphere area and assessment of cell death using DRAQ7 labelling. Peripheral blood mononuclear cells (PBMCs) for these experiments were obtained from healthy volunteers at the University Medical Centre Freiburg. Further information can be found in the Supplementary methods.

### qPCR analysis

For the quantitative assessment of gene expression, qPCR (quantitative polymerase chain reaction) analyses with the SYBR master mix plus have been used as already described [17].

For the specific primer sequences used in the qPCR analyses, please refer to Supplementary Table S2 for detailed information.

### Human monocyte culture and macrophages-like differentiation

Human peripheral blood samples were obtained from healthy donors and the monocytes were enriched with CD14 antibodies (Miltenyi, 130–050-201) by positive selection. The purified human monocytes were cultured in RPMI 1640 medium with Glutamax-I supplemented with 10% FBS. To induce macrophage differentiation, the purified monocytes were stimulated for 5 days with colony-stimulating factor 1 (CSF-1) to generate M0-like macrophages. To induce polarization into different macrophage phenotypes, the M0-like macrophages were subjected to specific treatments. For M1-like polarization, lipopolysaccharide (100 ng/ml) and interferon gamma (20 ng/ml) were administered to M0-like macrophages for 48 h. M2-like polarization was achieved by delivering IL-4 (20 ng/ml) to M0-like macrophages for 48 h. After polarization, M0-, M1- and M2-like macrophages were treated with RCT001 at a concentration of 2.5 µM applied 48 h after polarization. Further information can be found in the Supplementary methods.

### Flow cytometry analysis

In the context of analyzing human primary macrophages, flow cytometry was employed to characterize macrophage polarization and other specific markers. See Supplementary methods for more information.

### ***Tumor ***experiments

A total of 200,000 RENCA cells were subcutaneously inoculated into the flanks of female BALB/c mice obtained from Janvier Labs. When the tumors size reached approximately 30 mm^3^, the mice were categorized into distinct groups, each designated for a specific treatment regime as described in the figure: control group (InVivoMab, BX-BE0089), RCT001 groups (5 or 20 mg/kg), anti-PD-1 groups (100 μg, InVivoMab, BX-BE0146), anti-CTLA4 groups (100 μg, InVivoMab, BX-BE0164) and combination. The treatments were administered via intra-peritoneal injections spanning an 11-day period. RCT001 was administered daily, and the immune checkpoint inhibitors (ICIs) were administered three times a week. The efficacy of the treatments was evaluated based on the size and weight of the tumors. When the tumors reached an approximate size of 600 mm^3^, they were harvested, and their weights were measured.

### Study approval

Study approved by the Veterinary Service and the Direction of Sanitary and Social Action of Monaco.

#### Tumor dissociation & cytometry analysis

Briefly, the tumors are dissociated, then the isolated cells are labeled and cytometrically analyzed to quantify the different immune cell populations present within the tumor. See Supplementary methods for more information.

#### Immunohistochemistry

Paraffin-embedded RCC tumors were cut, and sections were subjected to an incubation with an α-SMA (alpha-smooth muscle actin, Agilent, GA611) antibody at a dilution of 1:500. Following the primary antibody incubation, a biotinylated secondary antibody from DAKO was applied to the tissue sections. The binding of the secondary antibody was detected using the diaminobenzidine (DAB) substrate. To enhance tissue visualization and contrast, a hematoxylin counterstain was applied.

### Patients

#### Ethics approval

The study exclusively involved adult patients, aligning with ethical considerations. This investigation adhered to the principles outlined in the Declaration of Helsinki, underscoring its commitment to ethical research conduct.

### Cohort

#### *Our *cohort

Samples of primary tumors were collected from patients diagnosed with ccRCC at Leuven University Hospital, Belgium. The focus of investigation was the expression of ELR + CXCLs mRNA. See Supplementary methods for more information.

#### Published* cohort*

Analysis of ELR^+^CXCLs mRNA Levels in ccRCC. The analyses in this study were based on data previously published in the article authored by Braun et al. [21] (Supplementary « 41591_2020_839_MOESM2_ESM»*).*

### Statistics

Calculation of PFS and OS involved patient subgroups based on ELR^+^CXCL mRNA levels that were either below or above the third quartile value. For PFS, the time span between initiation of systemic therapy and either disease progression or death from any cause was measured. In cases where patients were at the last follow-up and did not show progression, they were censored. OS covered the period from the start of systemic therapy until the date of death from any cause. Patients who were alive at the last follow-up were censored.

Survival curves were generated using the Kaplan–Meier method. Analyses that accounted for censored data were conducted utilizing Cox proportional hazards models. To carry out these analyses, the R software, version 3.2.2 (https://www.r-project.org/), was employed.

### Statistical analysis and data presentation

Statistical analyses of the data were conducted using GraphPad Prism 8 software. The results were presented as mean ± standard deviation (SD). For comparisons between two groups, an unpaired Mann–Whitney test was employed. For analyses involving multiple groups, a two-way analysis of variance (ANOVA) was used. To address the issue of multiple comparisons, a Bonferroni post-hoc test was applied.

## Results

### ELR^+^CXCLs expression is associated with poor OS in RCC patients treated by ICIs

**Fig. 1:**
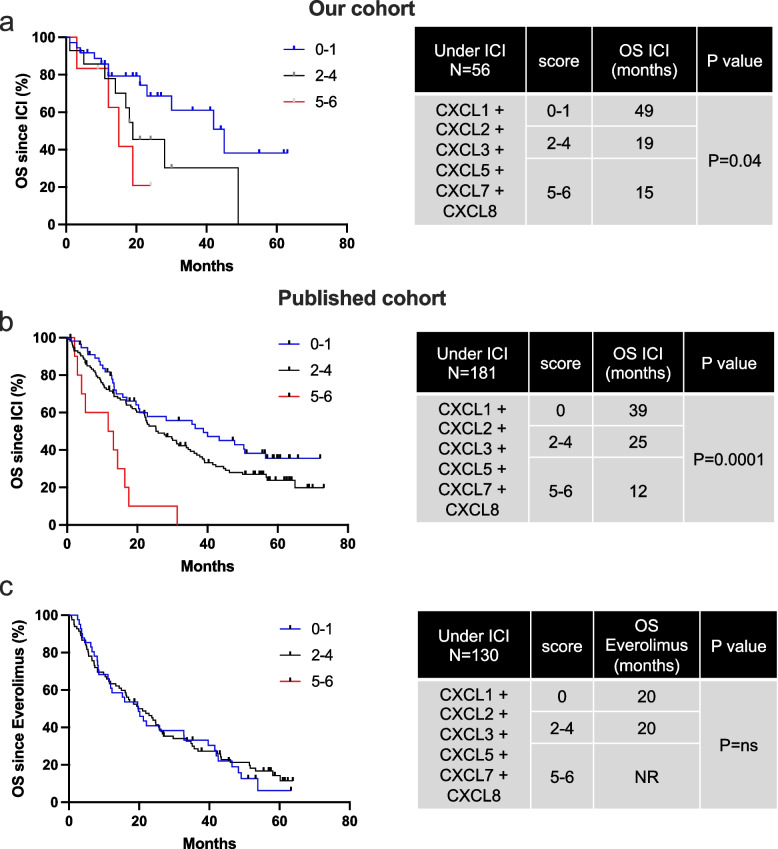
High ELR^+^CXCL expression is associated with poor OS under ICI in RCC patients. Levels of ELR^+^CXCLs (CXCL1, CXCL2, CXCL3, CXCL5, CXCL8) and PD-L1 mRNA expression in metastatic RCC patients on ICIs or everolimus were measured by RT-qPCR and correlated with OS in patients from our cohort (ICI treatment, 56 patients, **a**) and in published cohort (ICI treatment, 181 patients, **b** or everolimus, 130 patients, **c**). The value of the third quartile of ELR^+^CXCL expression was chosen as the cut-off value. We created a score reflecting the overexpression of each ELR^+^CXCL (score 0 = no ELR^+^CXCL overexpressed, score 6 = all 6 ELR^+^CXCL overexpressed). The score was correlated with OS. The Kaplan–Meier method was used to generate survival curves and analyses of censored data were performed using Cox models. Statistical significance (*p*-values) is indicated

We assessed the mRNA expression levels of ELR^+^CXCLs (CXCL1, CXCL2, CXCL3, CXCL5, CXCL7 and CXCL8) in tumor of ccRCC patients who received treatment with ICIs (nivolumab or nivolumab + ipilimumab, *n* = 237), as well as everolimus (Fig. 1a-c, Table 1, *n* = 130), with two distinct cohorts: our cohort (*n* = 56) and the published cohort (*n* = 181, [21]). This investigation aimed to ascertain the potential correlation between ELR^+^CXCLs expression and patient outcomes. Our analysis reveals that high expression levels (greater than the third quartile) of CXCL2, CXCL7 and CXCL8 were associated with a shorter OS among patients treated with ICIs (Table 1). Considering the overlapping and redundant roles of all ELR^+^CXCL cytokines, we devised a composite score representing the collective overexpression of each ELR^+^CXCL (score 0: no ELR^+^CXCL overexpressed, score 6: all 6 ELR^+^CXCL overexpressed). High ELR^+^CXCL expression (score 5–6) correlated with significantly lower OS in patients undergoing ICIs treatment (median OS of 15 vs 49 months in our cohort, and 12 vs 39 months in the published cohort, Fig. 1a-b). Although patients with overexpressed PD-L1 display a tendency towards extended OS, this predictive mRNA biomarker of ICI efficacy did not demonstrate statistical significance.

**Table 1:**
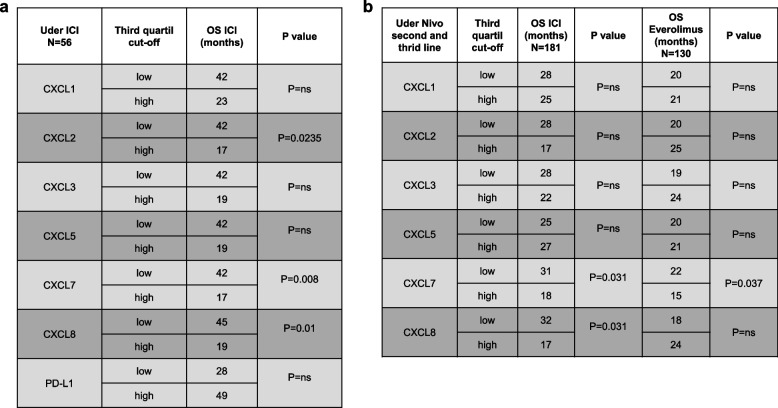
High ELR^+^CXCL expression is associated with poor OS under ICI in RCC patients. ELR^+^CXCLs levels (CXCL1, CXCL2, CXCL3, CXCL5, CXCL8) and PD-L1 mRNA expression in patients with metastatic RCC treated with ICIs or everolimus were measured by NGS and correlated with OS in patients from our cohort (ICI treatment, 56 patients, **a** and from the published cohort (ICI treatment, 181 patients, **b** or everolimus treatment, 130 patients, **c**. The third quartile value of CXCLs expression was chosen as the cut-off value and correlated with OS. The Kaplan–Meier method was used to generate survival curves and analyses of censored data were performed using Cox models. Statistical significance (*p*-values) is indicated

Similar results were observed in patients treated with TKIs (Fig. S1, Table S1, *n* = 100). In patients treated with everolimus, ELR^+^CXCL overexpression did not exhibit a correlation with OS (Fig. 1c). ELR^+^CXCL levels correlate with response to both ICIs and TKIs, indicating their importance in the mechanisms of action of both cornerstones of current RCC treatment.

This comprehensive analysis underscores the potential significance of ELR^+^CXCL expression levels as predictive markers of patient response to ICIs, providing valuable insights into therapeutic strategies for RCC patients.

### Development of CXCR2 Inhibitor: Hit-to-Lead Optimization towards the Identification of RCT001 as Lead Compound

Building upon previous work in the development of CXCR2 inhibitors [17], we have devoted efforts to refine the structure of our initial hit compound. This successive multiparametric optimization aimed at improving both the efficiency and the pharmacological profile of this family of *N,N’*-diarylurea compounds. Thus, focused series of derivatives were iteratively designed, synthesized, and biologically evaluated through different models to establish a structure–activity relationship. This allowed to define the crucial role of several substituents for the activity and to select our lead molecule, designated as RCT001 (molecular structure in Fig. S2; Grytsai et al., in revision). This novel compound displays improved pharmacological properties and a refined inhibitory profile, selective against CXCR2. This significant achievement in the evolution of our CXCR2 inhibitors underscores our dedication to translating scientific knowledge into tangible advancements in the field of drug discovery, and to advancing towards preclinical development of a therapeutic agent targeting CXCR2-mediated pathways.

### RCT001 is an inverse agonist of CXCR2: Mechanism of action and experimental validation

To comprehend the impact of RCT001 on CXCR2, we harnessed an innovative technology developed by G. CLIPS biotech. This approach involves the production of recombinant CXCR2 which is subsequently reconstituted in detergent buffers incorporating lipid composition akin to the receptor’s lipid environment. A non-modifying probe, facilitating the detection of receptor activation and conformational changes upon ligand binding, is employed. The emission spectra of this probe exhibit shift in maximum wavelength (λmax) corresponding to receptor activation or deactivation, enabling real-time monitoring λmax of ligand-induced changes.

**Fig. 2:**
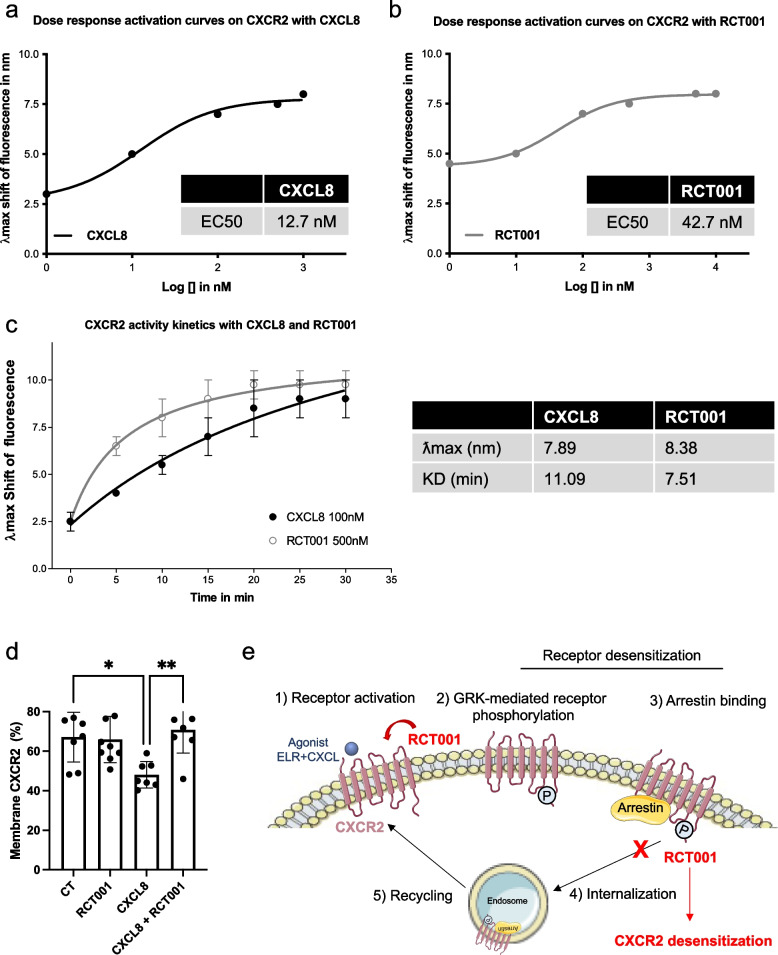
RCT001 is an inverse agonist of CXCR2. **a-c** The recombinant CXCR2 reconstituted in detergent buffer containing lipids that mimic the lipid composition of the receptor, is then labelled with a non-modifying probe that allow detection of the activation state and conformational change of the receptor upon addition of a ligand. The maximum wavelength of the probe emission spectrum (λmax) shifts according to the activation (TM6 opening)/inactivation (TM6 closing) of the receptor. The kinetics of receptor activation/inactivation can be followed by observing the λmax shift over time after addition of a ligand. **a** CXCR2 activation was measured by the λmax shift 20 min after stimulation by different doses of CXCL8. **b** CXCR2 activation was measured by the λmax shift 20 min after stimulation by a dose response of RCT001. **c** CXCR2 kinetic activation (0 to 30 min) was measured by the λmax shift after stimulation by CXCL8 (100 nM) or RCT001 (500 nM). **d** Starved endothelial cells were stimulated with 50 ng/ml CXCL8 for 1 h. Membrane-associated CXCR2 protein levels were quantified by flow cytometry. Results are presented as the mean of three independent experiments ± SD. Statistics were performed using the ANOVA test: *p < 0.05; **p < 0.01. **e** Summary schematic of the mechanism of action of RCT001 as an inverse CXCR2 agonist

Initial validation of the technology was executed with CXCR2, corroborating that its natural ligand, CXCL8, indeed acts as an agonist by activating CXCR2 (as indicated by a positive λmax shift). CXCR2 activation by CXCL8 showcased dose-dependent behavior (Fig. 2a). Subsequently, we explored RCT001’s impact on CXCR2. Surprisingly, RCT001 demonstrated similar effects as CXCL8 inducing receptor activation (evidenced by a positive λmax shift) (Fig. 2b). RCT001's impact on CXCR2 activation was also found to be dose-dependent, with respective EC50 values of 12.7 nM for CXCL8 and 42.7 nM for RCT001. Notably, RCT001 exhibited a more efficient induction of transmembrane domain 6 (TM6) of CXCR2 opening compared to CXCL8, reflected by faster binding kinetics (KD 7.51 vs. 11.09 min respectively, Fig. 2c). These results were confirmed using two additional ligands: CXCR2 ligands: CXCL1 and CXCL7 (Fig. S3a-b). CXCL1 exhibits rapid binding to CXCR2 similar to RCT001 (KD 6.5 min), while CXCL7, like CXCL8, binds CXCR2 slower than RCT001 (KD 9.06 min). To contrast with conventional antagonists, we examined the behavior of a known CXCR2 inhibitor, SX-682. Unlike RCT001, SX-682 inhibited CXCL8-induced pocket opening of CXCR2 (Fig. S3c), firmly establishing it as a standard CXCR2 antagonist. Given that CXCR2 internalization occurs upon ELR^+^CXCL stimulation, primarily contributing to receptor desensitization, we evaluated RCT001’s potential to inhibit CXCR2 internalization in endothelial cells and its resulting in the transfer of receptors from the plasma membrane to the endosomal compartment [22]. CXCL8 stimulation led to CXCR2 internalization (manifested as a reduction in % membrane CXCR2). In the presence of RCT001, CXCL8 failed to induce CXCR2 internalization, indicating that RCT001 prevented the CXCL8-dependent internalization process** (**Fig. 2d).

In summary, our findings reveal that RCT001, like CXCL8, induces the opening of CXCR2 pocket. However, RCT001 inhibits CXCR2 internalization, leading to receptor desensitization and rendering it incapable of activation.

In pharmacological terms, RCT001 can be categorized as an inverse agonist- an agent that binds to the same receptor as an agonist but triggers a response opposite to that of the agonist.

Given its distinct mechanism of action as an inverse agonist, RCT001 stands out as a unique and promising CXCR2 inhibitor, setting it apart from other agents within the CXCR2 inhibitor landscape.

### RCT001 inhibits in vitro angiogenesis and primary RCC cell viability

**Fig. 3:**
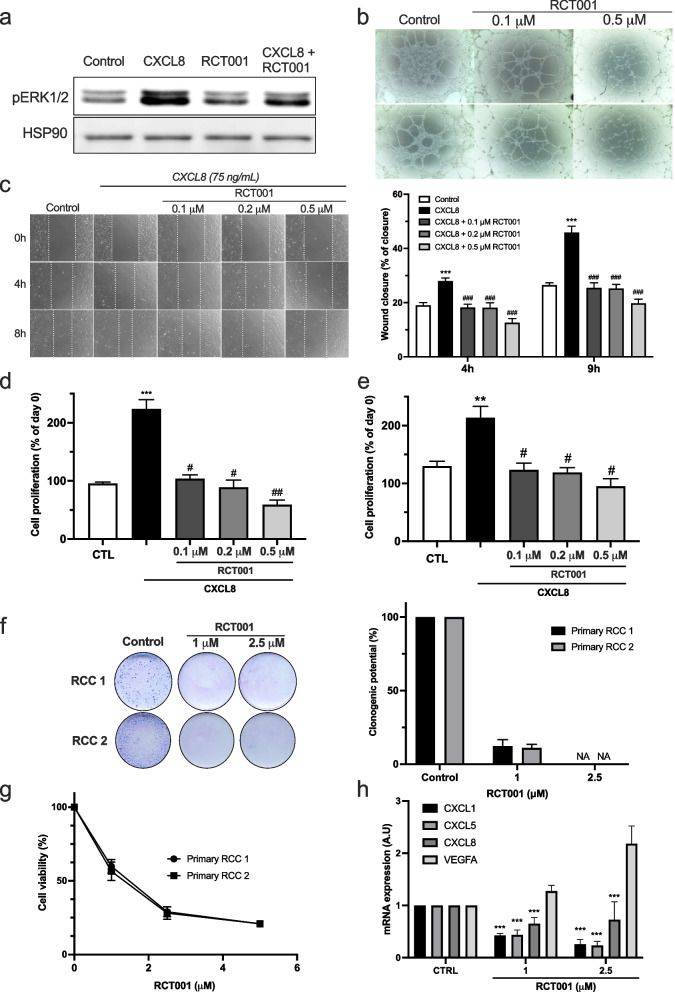
RCT001 inhibits angiogenesis and viability of primary RCC cells in vitro. **a** Immunoblot analysis of pERK1/2 expression in serum-depleted endothelial cells determined 24 h after stimulation by CXCL8 (75 ng/mL) and/or treatment with RCT001 (1 μM). **b** Matrigel-based tube formation assay was performed with endothelial cells 4 h after treatment with RCT001 (0.1 or 0.5 μM). **c.** Wound scratch assay was performed with serum-depleted endothelial cells treated or not with CXCL8 (75 ng/mL) in the presence of RCT001 (0.1, 0.2 or 0.5 μM). Wound closure was determined 4 and 8 h after treatment. **d-e** Cell proliferation assay of serum-depleted endothelial cells treated or not with CXCL7 (75 ng/mL) (**d**) or CXCL8 (75 ng/mL) (**e**) in the presence of RCT001 (0.1, 0.2 or 0.5 μM) for 72 h. **f** Clonogenic assay with two primary RCC cells in the presence or not of RCT001 (1 or 2.5 μM). **g** Viability of two primary RCC cells treated with RCT001 for 48 h. **h** Evaluation of CXCL1/5/8 and VEGFA mRNA levels in primary RCC cells after 24 h of treatment with RCT001 treatment (1 or 2.5 μM) by qPCR. Results are presented as the mean of three independent experiments ± SD. Statistics were performed using the ANOVA test: **p < 0.01, *** *p* < 0.001 vs control conditions, ^#^*p* < 0.01, ^###^*p* < 0.001 vs CXCL8 conditions

To comprehensively assess the dual impact of RCT001 on both endothelial (TIME cells) and two primary RCC tumor cells, we conducted a series of investigations. We began by corroborating our earlier findings, observing that CXCL8 treatment induced an upregulation of pERK1/2 expression in endothelial cells. This effect was effectively reversed upon treatment with RCT001 (Fig. 3a). This molecular alteration translated into an observable anti-angiogenic phenotype, as evidenced by subsequent assays (Fig. 3b-e).

**Fig. 4:**
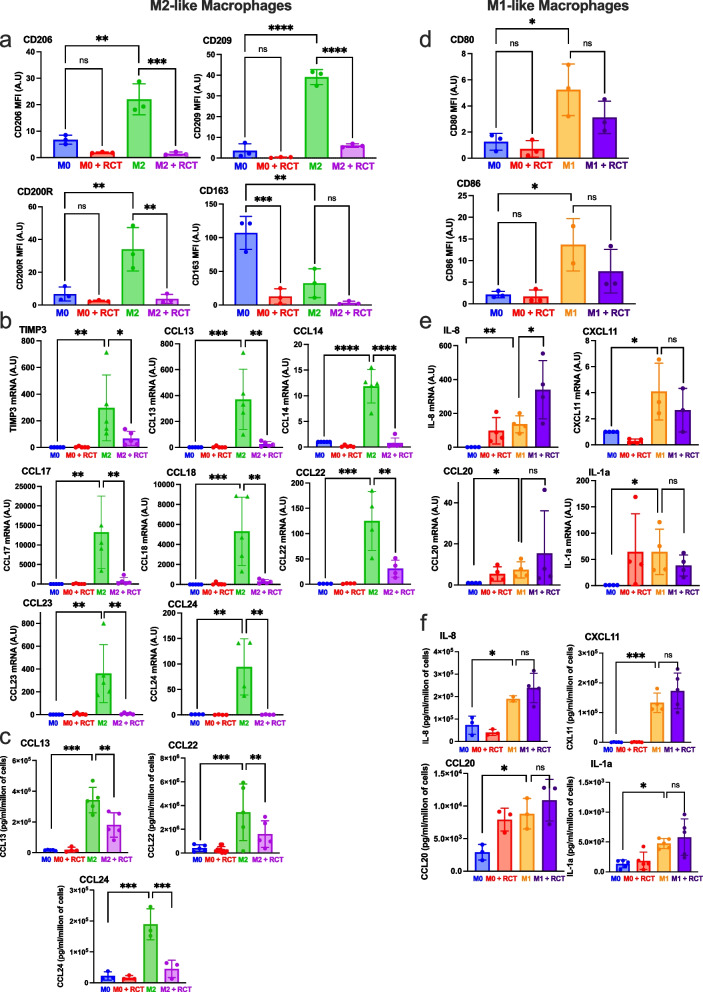
RCT001 reverses the polarization of M2-like macrophages polarization. Purified monocytes from healthy human donors (n = 5) were differentiated into M0-like macrophages (5 days with CSF1) and then polarized into M0-like macrophages (CSF1), or M2-like macrophages (IL4) or M1-like macrophages (LPS) for 48 h. The already polarized macrophages then were treated with RCT001 (2.5 µM) in the presence of the respective cytokines (CSF1 for M0, IL4 for M2 and LPS for M1 macrophages) for 48 h. MFI: Mean Fluorescence Intensity **a-c** M0 and M2-like macrophages were treated with RCT001 for 48 h. **a** Specific M2 macrophage membrane markers (CD206, CD209, CD200R, CD163) were examined by flow cytometry. **b** Specific M2 macrophage mRNA markers (TIMP3, CCL13, CCL14, CCL17, CCL18, CCL22, CCL23, CCL24) were evaluated by qPCR. c. Specific cytokines secreted by M2 macrophages (CCL13, CCL22, CCL24) were determined by ELISA. **d-f** M0 and M1-like macrophages were treated with RCT001 for 48 h. **d** Specific M1 macrophage membrane markers (CD80, CD86) were evaluated by flow cytometry. **e** Specific M1 macrophages mRNA markers (IL8, CXCL11, CCL20, IL-1a) were evaluated by qPCR. **f** Specific cytokines secreted by M1 macrophages (IL8, CXCL11, CCL20, IL-1a) were determined by ELISA. Results are presented as the mean of five independent experiments ± SD. Statistics were performed using the ANOVA test: *p < 0.05, **p < 0.01, *** *p* < 0.001

Our assessment of RCT001's anti-angiogenic activity commenced with an endothelial tube formation assay (Fig. 3b). Here, we noted that RCT001 effectively disrupted the formation of tube-like networks in a dose-dependent manner. Furthermore, wound closure experiments involving endothelial cells unveiled a pro-migratory effect triggered by CXCL8, which was completely abrogated following RCT001 treatment (*p* < 0.001 for RCT001 at doses from 0.1 to 0.5 µM vs. CXCL8 alone). Additionally, CXCL7/8 treatment resulted in a remarkable 200% proliferation of serum-starved endothelial cells, a response that was entirely suppressed by RCT001 treatment at concentrations as low as 0.1 µM (Fig. 3d-e). Notably, RCT001 exhibited more potent anti-proliferative effects compared to other commercially available CXCR2 inhibitors, regardless of the dose (Fig. S4a-b). Furthermore, we demonstrated the specificity of RCT001 for CXCR2. RCT001 inhibited CXCL7/8-induced endothelial cell proliferation but had no effect on VEGF-induced proliferation (Fig. S4c). Our evaluation extended to assessing RCT001's potential to inhibit in vitro tumorigenesis, a process examined in two primary ccRCC (Fig. 4f-g). Impressively, clonogenic potential and cell viability were completely ablated by RCT001 treatment, evident at micromolar concentrations. This anti-tumor efficacy was reaffirmed in the context of human RCC cell lines, including 786-O and A498 (Fig. S4d-g). Furthermore, mRNA analysis performed on primary RCC cells displayed a significant reduction in CXCL1, CXCL5, and CXCL8 expression levels following RCT001 treatment (Fig. 3h).

Conclusively, RCT001 emerges as a potent inhibitor of ELR + CXCL-dependent angiogenesis and an effective anti-proliferative agent targeting RCC cells, both in established cell lines and primary cells obtained from patients. This multifaceted action highlights the potential therapeutic significance of RCT001 in mitigating angiogenesis and impeding RCC cell growth.

### RCT001 reverts M2-like macrophages polarization

Given the elevated expression of ELR^+^CXCL/CXCR2 proteins in human primary macrophages, we set out to investigate RCT001's capacity to effectively modulate the polarization of macrophages-like towards both the anti-tumor M1 phenotype or the pro-tumor M2 phenotype (Fig. 4).

Monocytes were enriched from human peripheral blood samples were obtained from healthy donors. To induce macrophage differentiation, the purified monocytes were stimulated for 5 days with colony-stimulating factor 1 (CSF-1) to generate M0-like macrophages. To induce polarization into different macrophage phenotypes, the M0-like macrophages were subjected to specific treatments (LPS + INFg for M1-like polarization, IL-4 for M1-like polarization).

After verifying the absence of any toxicity of RCT001 on the different kind of macrophages-like (Fig. S5), we turned our attention to the molecule's ability to modulate ex vivo macrophage polarization. First, we showed that RCT001 blocks the induction of M2-like polarization in response to IL-4, reducing the expression of CD206, CD209, CD200R, and CD163 (Fig. S6a-b). This outcome was further confirmed through qPCR analysis of specific M2 macrophage markers (Fig. S6C).

Importantly, RCT001 did not appear to modify the polarization of M1-like macrophages, as indicated by the observation that the expression of M1 macrophage markers even showed signs of increase during RCT001 treatment (Fig. S6D).

Secondly, we proceeded to examine whether RCT001 could reverse an already established M2 phenotype after polarization (Fig. 4). Our findings indicated that RCT001 effectively shifted M2-like macrophages towards the M0-like phenotype, as judged by the reduction in CD206, CD209, CD200R, and CD163 expression (Fig. 4a). This result was corroborated through qPCR analysis of specific markers (TIMP3, CCL13, CCL14, CCL17, CCL18, CCL22, CCL23, and CCL24) (Fig. 4b). Further substantiating this, we confirmed the suppression of M2 macrophage-secreted cytokines CCL13, CCL22, and CCL24 by RCT001 treatment (Fig. 4c).

Importantly, the effect of RCT001 on M2-like macrophage polarization was confirmed using specific siRNA against the CXCR2 receptor (Fig. S7). Indeed, the specific inhibition of CXCR2 expression is enough to block the induction of IL-4-mediated M2-like macrophage polarization. To solidify the specificity of the observed effects on macrophage polarization, we examined RCT001's impact on M1-like macrophages. Encouragingly, RCT001 did not alter the expression of M1 macrophage markers CD80 and CD86 analyzed through cytometry (Fig. 4d), nor did it influence the mRNA expression (Fig. 4e) and secretion (Fig. 4f) of M1 macrophage-specific cytokines IL-8, CXCL11, CCL20, and IL1α.

Collectively, our results underscore RCT001's potential to inhibit and reprogram the polarization of pro-tumor M2-like macrophages while leaving the anti-tumor M1-like macrophages unaffected. These insights augments RCT001's promise as a valuable anti-tumor agent, capable of targeting both tumor and stromal cells, including endothelial and immune cells, and potentially synergizing with ICI therapies to enhance their effectiveness.

### RCT001 efficiently inhibits the growth of experimental ccRCC, synergizes with ICIs and enhances immune reactivity in tumors

**Fig. 5:**
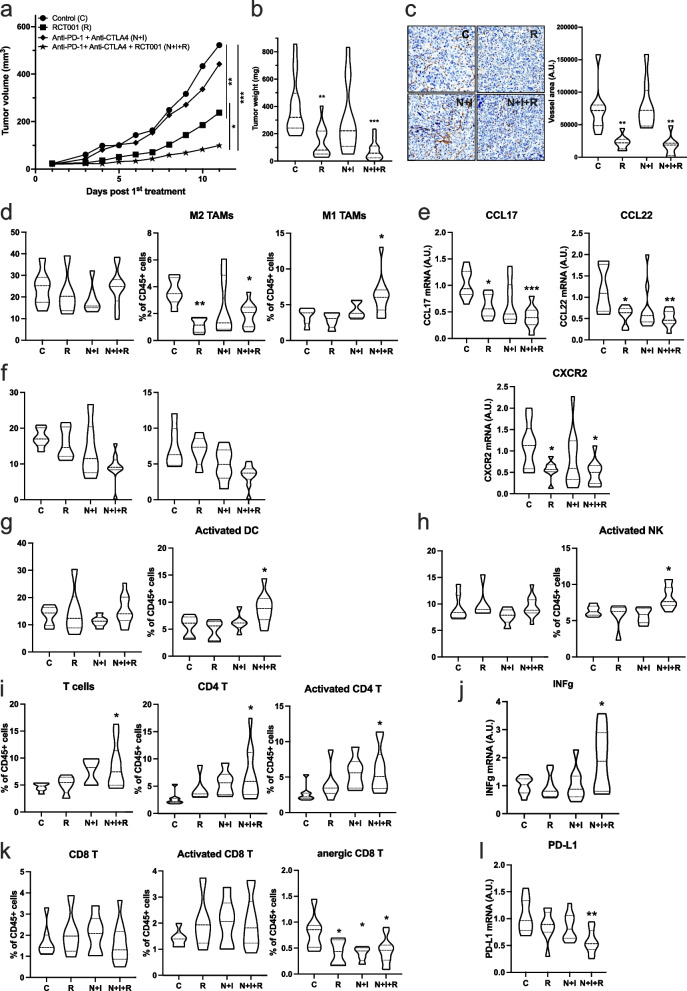
RCT001 efficiently inhibits RCC growth and synergizes with ICIs. RENCA cells were injected subcutaneously. Once the tumor reached 30 mm^3^, mice were divided into different groups: Control (*n* = 10), RCT001 alone (*n* = 10, 20 mg/kg), anti-PD-1 + anti-CTLA4 (*n* = 10, 100 μg each) and the combination of RCT001 + PD-1 + anti-CTLA4 (*n* = 10). **a** Tumor volume was measured each day. Results are expressed as mean ± sd. **b** Tumor weights at the end of the experiment. **c** Blood vessels were visualized and quantified by IHC for αSMA **d** Tumor-associated macrophages (TAMs, total population), M2 TAMs and M1 TAMs were evaluated by cytometry. **e** The specific mRNA markers of M2 TAMs (CCL17 and CCL22) and mRNA CXCR2 were determined by qPCR. **f** Tumor-associated neutrophils (TANs, total population) and mature TANs were evaluated by cytometry. **g** Intratumor dendritic cells (DCs, total population) and activated dendritic cells were evaluated by flow cytometry. **h** Intratumor natural killer (NKs, total population) and activated natural killer were evaluated by cytometry. **i** Intratumor T cells (total population) and CD4 + T, Activated CD4 + T were evaluated by flow cytometry. **j** Intratumor activated T cell specific mRNA marker (INFγ) mRNA was evaluated by qPCR. **k** Intratumor CD8 T, activated CD8 T and anergic CD8 T were evaluated by flow cytometry. **l** Intratumor anergic T cell specific mRNA marker (PD-L1) mRNA was evaluated by qPCR. Statistics were performed using the ANOVA test: *p < 0.01, **p < 0.01, *** *p* < 0.001

Given the enduring anti-tumor response witnessed with nivolumab and ipilimumab in clinical contexts, and the notable efficacy of RCT001 observed in M2 macrophages, we pursued an evaluation of RCT001 in combination with immune checkpoint inhibitors (ICIs) anti-PD-1 (like nivolumab) and anti-CTLA4 (like ipilimumab), against experimental RCC, as depicted in Fig. 5. RENCA cells were subcutaneously inoculated into the flanks of BALB/c mice. When the tumors size reached approximately 30 mm^3^, the mice were specific treatment regime as described in the figure.

Intriguingly, while nivolumab/ipilimumab therapy failed to hinder tumor growth in comparison to control conditions (as already described in this model), administration of RCT001 alone (20 mg/kg) notably delayed tumor progression (*p* < 0.01 vs control conditions or nivolumab/ipilimumab, Fig. 5a). Moreover, the convergence of both RCT001 and nivolumab/ipilimumab treatments produced an even more pronounced therapeutic effect, outperforming RCT001 monotherapy and control conditions (*p* < 0.05 and *p* < 0.001, respectively, Fig. 5a). This trend was further corroborated through ex vivo analyses, which exhibited the lowest tumor weights within the RCT001 monotherapy (60% reduction) and nivolumab/ipilimumab combined with RCT001 (80% reduction) groups, compared to the control (*p* < 0.01 and *p* < 0.001, respectively, Fig. 5b).

Furthermore, we have confirmed that inhibition of CXCR2 by RCT001, as described above, induces a decrease in blood vessels within tumors (Fig. 5c).

Because anti-PD-1 are also used in clinical practice alone (without anti-CTLA4) in second- and third-line treatment, we analyzed the efficacy of RCT001 in combination with anti-PD-1. As previously, anti-PD-1 treatment is not effective, but its combination with RCT001 enhances its efficacy (Fig. S8A). Nevertheless, the anti-PD-1 + anti-CTLA4 + RCT001 combination was more effective than the anti-PD-1 + RCT001 combination (Fig. S8A). To demonstrate the synergistic effect of RCT001 and anti-PD-1, we used a suboptimal dose of RCT001 (5 mg/kg), which had no effect on tumor growth. Nevertheless, in combination with anti-PD-1, RCT001 sensitizes tumors to anti-PD-1 (Fig. S8b).

Subsequently, we delved into the characterization of immune cell infiltration within RENCA tissue, specifically in response to triple combination therapy. To elucidate the precise composition of immune cell infiltrates within the tumor, we harnessed the analytical prowess of fluorescence-activated cell sorting (FACS) analysis. By unraveling the nature of these infiltrated immune cells, we could shed light on the immunological dynamics underlying the therapeutic outcomes.

Remarkably, the administration of RCT001 does not affect the total TAMs but decreases the pro-tumor M2 TAMs, as demonstrated on primary human macrophages (Fig. 5d). The decrease in the percentage of M2 TAMs visible by cytometry was also confirmed by the decrease in CCL17 and CCL22 mRNA (M2 macrophages markers), as well as CXCR2 mRNA in the tumor (Fig. 5e). RCT001 in synergy with anti-PD-1 and anti-CTLA4 yielded a substantial increase of M1 TAMs (Fig. 5d), a decreased of TANs (Tumor Associated Neutrophils) and mature TANs (associated with poor prognosis in RCC, Fig. 5f), accompanied by an increase in activated dendritic cells (DCs, Fig. 5g) and activated natural killer (NKs, Fig. 5h). The decrease in M2 TAMs and TANs (immunosuppression), associated with an increase in antigen presentation by DCs and NKs in the triple-treatment group, favored an increase in CD4 + T cells and activated CD4 + T cells (Fig. 5i), as well as overexpression of INFγ mRNA (Fig. 5j). Interestingly, no discernible differences were detected concerning CD8 + T cells or activated CD8 + T cells (Fig. 5k). However, a noteworthy elevation in the population of intratumor anergic CD8 + T cells was observed (Fig. 5k), accompanied by a decrease in PD-L1 mRNA (Fig. 5l).

Incorporating these findings into the broader context of our study, a coherent picture emerges. The collective evidence conclusively underscores that the therapeutic synergy achieved through combination therapy effectively fosters the infiltration of T cells, primarily CD4 + and activated CD4 + T cells, into the tumor microenvironment. This infiltration pattern aligns seamlessly with the observed robust anti-tumor effects. As such, our comprehensive results provide a compelling rationale for the remarkable potential of combination therapy in orchestrating the recruitment of critical immune cell players, thereby forging a promising avenue toward enhanced therapeutic outcomes in the context of RCC.

### RCT001 inhibits the proliferation of human RCC tumorspheres and synergizes with pembrolizumab

Following this, we proceeded to assess the efficacy of RCT001 in an in vitro model using Patient-Derived Xenograft (PDX) of human RCC, the RXF-486 from Charles River. Our objective was twofold: to validate RCT001's efficacy and its potential in combination with anti-PD-1 treatment within the context of primary RCC and the human immune system.

This PDX model is recognized for its expression of CXCR2 as well as ELR^+^CXCL cytokines such as CXCL1, CXCL2, CXCL5, and CXCL8 [23]. It is important to note that this model is inherently resistant to anti-PD-1 treatment, as referenced in prior studies.

**Fig. 6:**
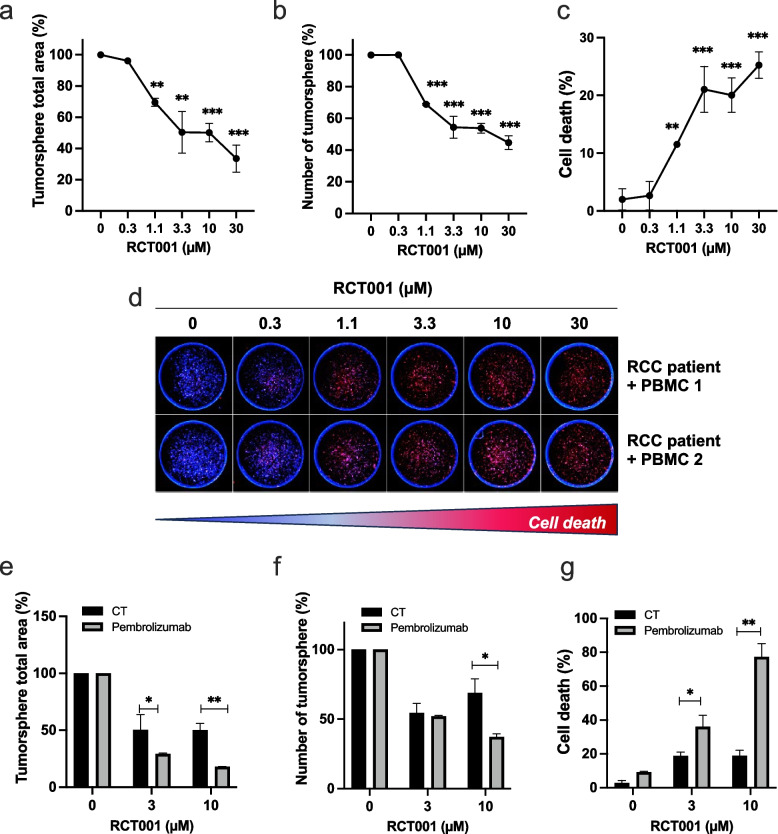
RCT001 inhibits proliferation of human RCC tumor spheres and synergize with anti PD-1. MEXF486 RCC tumors were grown as subcutaneous tumor xenografts in immunodeficient mice. After removal, the tumors are placed in layers of Versagel® from Cypre to achieve spheroid formation with human dermal fibroblasts (HDF). After the 3D tumors are established (7 days), they are treated with RCT001 (0.3 to 30 µM) for 7 days. **a** The total area of tumor spheroids was determined. **b** The number of tumor spheroids was determined. **c—d** Cell death was analyzed by DRAQ7 labelling. Quantification (**C**) and representative images (**D**) were shown. **e–g** Activated human PBMCs are first added to the cell culture medium (1 day before treatment); over time, they gradually infiltrate and attack the tumor spheres in the gel. Then tumor spheres + PBMCs were treated with RCT001 (3.3 and 10 µM) ± anti- PD -1 (pembrolizumab, 100 µg/ml). **e** The total area of tumorspheres was determined. **f** The number of tumorspheres was determined. **g** Cell death was analyzed by DRAQ7 labelling

In this experimental setup, after obtaining tumor samples (PDX), the tumor cells were cultivated within layers of Cypre's VersaGel®, a substrate that supports the formation of 3D tumorspheres. As previously, we calculated the IC_50_, defined as the concentration at which there is a 50% reduction in the total area of the tumorspheres is observed. Intriguingly, the IC_50_ value for RCT001 was determined to be 1.7 µM (Fig. 6a), signifying a significant reduction in tumorsphere area. Moreover, RCT001 administration led to a reduction in the number of tumorspheres (Fig. 6b) and notably induced cell death, achieving about 25% cell death at a concentration of 3.3 µM (Fig. 6c-d).

For the subsequent phase, our focus was on exploring the potential synergistic effects between RCT001 and anti-PD-1 treatment (pembrolizumab). In this pursuit, we introduced activated human Peripheral Blood Mononuclear Cells (PBMCs) to the experimental setup. Interestingly, the introduction of anti-PD-1 treatment alone did not induce cell death or significantly reduce the tumorsphere area (Fig. 6e-f). However, when RCT001 and anti-PD-1 were administered concomitantly, compelling results emerged. The combined treatment regimen led to a noteworthy reduction in the tumorsphere area (Fig. 6e), a decrease in the number of tumorspheres (Fig. 6f), and a substantial increase in cell death. Strikingly, when RCT001 was paired with anti-PD-1 at a concentration of 10 µM, cell death exceeded an impressive 70% (Fig. 6g).

In conclusion, our study using a human ccRCC PDX model enriched with primary ccRCC, and a functional human immune system confirms RCT001's strong efficacy. It also highlights the potential of combining RCT001 with anti-PD-1 treatment to boost anti-tumor responses and address resistant RCC challenges.

## Discussion

The therapeutic landscape of ccRCC has witnessed remarkable intensity and innovation over the past decade. Despite significant therapeutic advancements, achieving a definitive cure remains elusive. Among the notable improvements, anti-angiogenic drugs (TKI) and ICI stand out, extending survival by several years. The current challenge revolves around synergizing these treatments. Two approved combination approaches have demonstrated efficacy: the combination of independent ICIs (primarily anti-PD-1 plus anti-CTLA4) and ICI plus TKI, targeting the VEGF/VEGFR pathway. However, due to the considerable heterogeneity [24] of metastatic ccRCC, some metastases inevitably progress (excepted for very rare case).

Therefore, understanding the mechanisms that lead to relapse after immunotherapies is an important challenge. However, it was important to summarize and integrate the different therapies that have been developed in recent years to find out which different pathways lead to the ultimate goal of curing metastatic ccRCC. On our journey, we became more and more convinced of the importance of chronic inflammatory processes and their detrimental effects on the tumor microenvironment. These adverse effects were particularly significant for the response to immunotherapies.

We previously identified interconnected mechanisms/partners for resistance to TKI, including lysosome trapping [25], EGF receptor activation via down-regulation of PTPRκ [26], development of an alternative lymphatic network through VEGFC overexpression [27]. These mechanisms vary based on drug exposure time and entail adaptations by tumor cells and microenvironment components notably endothelial [28]. For instance, ccRCC exhibits an exacerbated inflammatory profile, where ELR^+^CXCL cytokines play a pivotal role in angiogenesis and inflammatory processes, including neutrophil attraction [15].

This initial discovery was made under the prism of superfluous angiogenic factors but their major role in chronic inflammation was key to resistance to ICIs. The initial immune cell attraction gradually transitions into chronic inflammation by polarizing toward myeloid-derived suppressor cells and M2 macrophages, producing pro-angiogenic/pro-inflammatory cytokines like ELR^+^CXCL [29].

Our main question was therefore to determine whether a treatment targeting angiogenesis and chronic inflammation could reveal specific pathway involved in resistance mechanisms to immunotherapies that is independent of the well-described VEGFA pathway. Such dual involvement in angiogenesis and immune tolerance prompted assessment in immunocompetent mice with ccRCC models insensitive to immunotherapies. Our study, the first to combine a CXCR2 inhibitor with ICI for the treatment of ccRCC, demonstrates that targeting CXCR2 not only inhibits angiogenesis, as observed in ccRCC xenografts [17], but also balances M2/M1 TAMs ratios. Furthermore, it enhances infiltration of NK cells, DCs and finally activation of CD4 + T cells. This immune cell influx likely contributes to RCT001's activity, alongside its effects on blood vessels. This outcome aligns with the heightened efficacy of combining nivolumab and ipilimumab in M1 macrophage-infiltrated tumors [30]. The robustness of our findings is underscored by experiments involving immunocompetent mice and PDX models supplemented with human PBMCs. A systematic exploration of structure–activity relationships guided us towards the discovery of a promising lead compound named RCT001. Through our investigations, we established that RCT001 functions as a CXCR2 inverse agonist, exhibiting an IC_50_ value of 45 nM specifically against CXCR2. While RCT001 displays a lower affinity for CXCR1 (IC_50_ in the µM range, data not provided), an intriguing observation emerged: after an 8 h treatment, RCT001 triggered a significant reduction in both CXCR2 expression at the mRNA and protein levels. This effect creates a "double" blockade of the signaling pathway upstream of these receptors.

Presently, other inhibitors targeting CXCR2 are undergoing clinical trials for diverse diseases. Prominent among these inhibitors are AstraZeneca's AZD5069 (for asthma and COPD), Syntrix Pharmaceutical's SX-682 (for skin melanoma and myelodysplastic syndrome), and Dompé Pharmaceutical's reparixin and ladarixin (for asthma and breast cancer). These inhibitors function through distinct mechanisms of action when compared to our compounds; notably, they do not operate as inverse agonists. Moreover, our investigations have demonstrated superior in vitro and in vivo efficacy of our inhibitor compared to these alternative inhibitors, suggesting a more potent therapeutic potential for RCT001.

Fibrosis has emerged as a pivotal mechanism driving the aggressiveness of ccRCC and ICI resistance, representing both a precancerous state and a contributor to heightened aggressiveness, especially considering the adverse prognosis associated with sarcomatoïd features [31]. This connection highlights the intricate relationship between fibrosis and disease progression. CXCR2 inhibitors have demonstrated their ability to attenuate fibrosis in experimental models of pancreatic tumors [32]. While we anticipate that RCT001 might exert analogous effects on fibrosis, there are certain limitations to its evaluation within our experimental tumor models. Specifically, the RENCA model lacks a fibrotic component, making it challenging to directly address the impact of RCT001 on fibrosis within this context.

Despite this limitation, the connection between CXCR2 inhibition and fibrosis reduction observed in other models, supports the hypothesis that RCT001 could indeed influence fibrotic processes in relevant settings. This avenue of research could potentially uncover further therapeutic dimensions for RCT001 in combating ccRCC's aggressive characteristics and its associated fibrosis-related challenges. In conclusion, our study unequivocally demonstrates that RCT001 significantly enhances the efficacy of ICIs in experimental models of ccRCC.

This distinction in mechanisms of action and enhanced efficacy reinforces the unique position of RCT001 as a promising candidate in the landscape of CXCR2 inhibition therapy. Our findings highlight the potential of RCT001 to offer novel avenues for addressing diseases that involve CXCR2 signaling pathways, underscoring its value in therapeutic innovation.

### Supplementary Information

**Additional file 1. Supplementary Table S1.** High ELR^+^CXCL expression is associated with poor PFS and OS on TKI in RCC patients. The levels of ELR^+^CXCLs (CXCL1, CXCL2, CXCL3, CXCL5, CXCL8) mRNA expression in metastatic RCC patients under TKI (sunitinib, axitinib) first-line treatment were measured by NGS. The value of the third quartile of ELR^+^CXCL expression was chosen as the cut-off value and correlated with PFS and OS. The Kaplan-Meier method was used to generate survival curves and analyses of censored data were performed using Cox models. The statistical significance (*p*-values) is given. **Supplementary Table S2.** Primer sequences used in the qPCR analyses. “m” for mouse primer. **Supplementary Table S3.** Antibody used for immune cells characterization in tumor. **Supplementary Figure S1.** High ELR^+^CXCL expression is associated with poor PFS and OS on TKI in RCC patients. The levels of CXCLs (CXCL1, CXCL2, CXCL3, CXCL5, CXCL8) mRNA expression in metastatic RCC patients under first-line TKI (sunitinib, axitinib) treatment were measured by NGS. The value of the third quartile value of CXCLs expression was chosen as the cut-off value. We created a score reflecting the overexpression of each ELR^+^CXCL (score 0= no ELR^+^CXCL overexpressed, score 6= all 6 ELR^+^CXCL overexpressed). The score was correlated with PFS (a) and OS (b). The Kaplan-Meier method was used to generate survival curves and analyses of censored data were performed using Cox models. Statistical significance (*p*-values) is indicated. **Supplementary Figure S2.** RCT001 synthesis and characterization. Protocol for the synthesis of RCT001. ^1^H NMR spectrum and HPLC chromatogram assessing the purity of the compound > 99.9%. **Supplementary Figure S3.** SX-682 is a classical antagonist of CXCR2. a-b. CXCR2 kinetic activation (0 to 30 min) was measured by the λmax shift after stimulation by RCT001 (500 nM), CXCL1 (100 nM, a) or CXCL7 (100 nM, b) c. CXCR2 Kinetic activation (0 to 60 min) was measured by the λmax shift after stimulation by CXCL8 (100nM), and 30 min after treatment with the CXCR2 inhibitor SX-682 (500 nM, MedChemExpress, #HY-119339). **Supplementary Figure S4.** RCT001 inhibits angiogenesis and viability of RCC cells *in vitro*. a. Cell proliferation assay of serum-depleted endothelial cells treated with CXCL8 (75 ng/mL) in the presence of RCT001 (0.1 mM) or SX682 (CXCR2 inhibitor, 0.1 and 10 µM, MedChemExpress, #HY-119339) or ladarixin (CXCR2 inhibitor, 0.1 and 10 µM, MedChemExpress, #HY-19519) for 72 h. c. Cell proliferation assay of serum-depleted endothelial cells treated with VEGF (75 ng/mL) in the presence of RCT001 (0.1 to 0.5 mM) for 72h. d-e. RCC cell viability A498 (d) and 786-O (e) treated with RCT001 for 48 h. f. Immunoblot analysis of the expression of pAKT and pERK1/2 in RCC cells treated with RCT001 (2.5 mM) for 24 or 48 h. g. CXCL8 (75 ng/ml) dependent migration of RCC cells was analyzed using Boyden chamber assays in the presence/absence of RCT001 (0.1, 0.25 or 0.5 µM). Results are presented as the mean of three independent experiments ± SD. Statistics were performed using the ANOVA test: **p < 0.01, *** *p* < 0.001 vs control conditions, ^#^*p*< 0.01, ^###^*p* < 0.001 vs CXCL8 conditions, ^&^*p*< 0.05, ^&&&^*p* < 0.001 vs CXCL 8+ 0.1µM RCT001 conditions. **Supplementary Figure S5.** RCT001 has no effect on macrophage-like viability. M0, M1 or M2-like macrophages were treated with RCT001 (2.5 µM) for 48 h. Cell death was determined by cytometry. Results are presented as the mean of three independent experiments ± SD. Statistical significance was determined using an unpaired t-test. **Supplementary Figure S6.** RCT001 inhibits polarization of M2-like macrophages. Purified monocytes from healthy human donors (*n*=4) were differentiated into M0-like macrophages (5 days with CSF1) and then polarized into M0-like macrophages (CSF1), or M2-like macrophages (IL4) or M1-like macrophages (LPS) for 48 h, in the presence/absence of RCT001 (2.5µM). a-c. M0-like macrophages were polarized into M0 or M2-like macrophages in the presence of RCT001. a-b. Specific M2 macrophage membrane-markers (CD206, CD209, CD200R, CD163) were evaluated by flow cytometry. c. Specific M2 macrophage mRNA markers (TIMP3, CCL17, CCL18, CCL22, CCL23, CCL24) were evaluated by qPCR. d. M0-like macrophages were polarized into M0 or M1-like macrophages in the presence of RCT001. Specific M1 macrophage mRNA markers (CXCL11, CCL20) were evaluated by qPCR. Results are presented as the mean of four independent experiments ± SD. Statistics were performed using the ANOVA test: *p < 0.05, **p < 0.01, *** *p* < 0.001. **Supplementary Figure S7.** Reduction of CXCR2 expression inhibits M2-like macrophages polarization. Purified monocytes from healthy human donors were differentiated into M0-like macrophages (5 days with CSF1) and then transfected with siRNA against CXCR2 (siCXCR2) or control (siCT) for 48 h and polarized into M2-like macrophages (IL4) for 48 h. Specific M2 macrophage membrane markers (CD206, CD209, CD200R, CD163) were evaluated by cytometry. Results are presented as the mean of three independent experiments ± SD. Statistics were performed with an unpaired *t-*test: *p < 0.05,*** *p* < 0.001. **Supplementary Figure S8.** RCT001 efficiently inhibits RCC growth and synergizes with ICIs. RENCA cells were injected subcutaneously. Once tumor reached 30 mm^3^, mice were divided into different groups. Tumor volume was measured every day. The results are given as the mean values ± sd. a. Control (*n*=10), RCT001 alone (n=10, 20 mg/kg), anti-PD-1 (*n*=10, 100 mg) and the combination of RCT001 + PD-1 (*n*=10). b. Control (*n*=10), RCT001 suboptimal concentration (*n*=10, 5 mg/kg), anti-PD-1 (*n*=10, 100 mg) and the combination of RCT001+ PD-1 (n=10). Statistics were performed using the ANOVA test: *p < 0.01, **p < 0.01, *** *p* < 0.001.

**Additional file 2.**

## Data Availability

The datasets used and/or analyzed during the current study available from the corresponding authors on reasonable request. Maeva.dufies@gmail.com or gilles.pages@unice.fr.

## Abbreviations

ccRCC: Clear cell renal cell carcinoma
CXCR2: C-X-C chemokine receptor 2
HDF: Human Dermal Fibroblast
ICI: Immune checkpoint inhibitors
M1 ccRCC: Metastatic ccRCC
OS: Overall survival
PBMC: Peripheral blood mononuclear cells
PFS: Progression-free survival
RCC: Renal cell carcinoma
TAM: Tumor-associated macrophage
TKI: Tyrosine kinase inhibitors
VEGF: Vascular endothelial growth factor
α-SMA: Alpha-smooth muscle actin
