# Supplementary material for: Unveiling CXCR2 as a promising therapeutic target in renal cell carcinoma: exploring the immunotherapeutic paradigm shift through its inhibition by RCT001
Source: J Exp Clin Cancer Res. 2024 Mar 19;43:86. doi: 10.1186/s13046-024-02984-2 (PMC10949812; doi:10.1186/s13046-024-02984-2)
Supplement: Supplementary file 1 — Additional file 1. Supplementary Table S1. High ELR+CXCL expression is associated with poor PFS and OS on TKI in RCC patients. The levels of ELR+CXCLs (CXCL1, CXCL2, CXCL3, CXCL5, CXCL8) mRNA expression in metastatic RCC patients under TKI (sunitinib, axitinib) first-line treatment were measured by NGS. The value of the third quartile of ELR+CXCL expression was chosen as the cut-off value and correlated with PFS and OS. The Kaplan-Meier method was used to generate survival curves and analyses of censored data were performed using Cox models. The statistical significance (p-values) is given. Supplementary Table S2. Primer sequences used in the qPCR analyses. “m” for mouse primer. Supplementary Table S3. Antibody used for immune cells characterization in tumor. Supplementary Figure S1. High ELR+CXCL expression is associated with poor PFS and OS on TKI in RCC patients. The levels of CXCLs (CXCL1, CXCL2, CXCL3, CXCL5, CXCL8) mRNA expression in metastatic RCC patients under first-line TKI (sunitinib, axitinib) treatment were measured by NGS. The value of the third quartile value of CXCLs expression was chosen as the cut-off value. We created a score reflecting the overexpression of each ELR+CXCL (score 0= no ELR+CXCL overexpressed, score 6= all 6 ELR+CXCL overexpressed). The score was correlated with PFS (a) and OS (b). The Kaplan-Meier method was used to generate survival curves and analyses of censored data were performed using Cox models. Statistical significance (p-values) is indicated. Supplementary Figure S2. RCT001 synthesis and characterization. Protocol for the synthesis of RCT001. 1H NMR spectrum and HPLC chromatogram assessing the purity of the compound > 99.9%. Supplementary Figure S3. SX-682 is a classical antagonist of CXCR2. a-b. CXCR2 kinetic activation (0 to 30 min) was measured by the λmax shift after stimulation by RCT001 (500 nM), CXCL1 (100 nM, a) or CXCL7 (100 nM, b) c. CXCR2 Kinetic activation (0 to 60 min) was measured by the λmax shift after stim [file 13046_2024_2984_MOESM1_ESM.pdf]

Our cohort

Under TKI first line N=100		PFS TKI (months)	P value	OS TKI (months)	P value
CXCL1	low	9	P=0.0418	32	P=0.0409
	high	3		18	
CXCL2	low	8	P=ns	32	P=0.08
	high	6		23	
CXCL3	low	8	P=ns	32	P=ns
	high	7		30	
CXCL5	low	10	P=0.0003	38	P=0.0046
	high	3		25	
CXCL7	low	10	P=0.008	37	P=ns
	high	3		23	
CXCL8	low	8	P=ns	34	P=0.0235
	high	4		18	

Supplementary Table S1

	Forward	Reverse
**CCL17**	5' TTCCCCTTAGAAAGCTGAAG	5' CTTCACTCTCTTGTTGTTGG
**CCL18**	5' CTATACCTCCTGGCAGATTC	5' CTCTCTTGGTTAGGAGGATG
**CCL20**	5' TATATTGTGCGTCTCCTCAG	5' GCTATGTCCAATTCCATTCC
**CCL23**	5' TGGAGAGTTACTTTGAAACG	5' ACAAGTTCAATTCTTCCTGG
**CCL24**	5' TAGTAACCAGCCTTCTGTTC	5' CTCAGGAATTCTCTTGGAAAC
**CXCL1**	5' ATGCTGAACAGTGACAAATC	5' TCTTCTGTTCCTATAAGGGC
**CXCL11**	5' CTACAGTTGTTCAAGGCTTC	5' CACTTTCACTGCTTTTACCC
**CXCL5**	5' GATGAGTACAAAAGTCCTGATCCA	5' CTGCAGCCACTGGTTCTGT
**CXCL8**	5' GTTTTTGAAGAGGGCTGAG	5' TTTGCTTGAAGTTTCACTGG
**CXCR2**	5' CCAGTCAGGATTTAAGTTTACC	5' GTTGATTTCCAGGGATTCTG
**IL-1a**	5' AGAGGAAGAAATCATCAAGC	5' TTATACTTTGATTGAGGGCG
**mCCL17**	5' CATTCCTATCAGGAAGTTGG	5' CAGTCAGAAACACGATGG
**mCCL22**	5' CACATAACATCATGGCTACC	5' CAGAAGAACTCCTTCACTAAAC
**mCXCR2**	5' CTACTGCAGGATTAAGTTTACC	5' GACGTATATTACAACCACAGC
**mPD-L1**	5' CAAGTGAGAATGCTAGATGTG	5' TCCATCTTGAGTCTTTGGAC
**PD-L1**	5'-ATGCCCCATACAACAAAATC	5'-GACATGTCAGTTCATGTTCAG
**TIMP3**	5' CATGTGCAGTACATCCATAC	5' AGGTGATACCGATAGTTCAG
**VEFGA**	5' TTTCTGCTGTCTTGGGTGCATTGG	5' ACCACTTCGTGATGATTCTGCCCT

Supplementary Table S2

	Panel	Channel	Suppliers	References
**CD107**	1	FITC	Biolegend	121605
**CD11b**	2	BV605	Biolegend	101257
**CD11c**	2	BV785	Biolegend	117335
**CD163**	2	PE-eFluor 610	Invitrogen	61-1631-80
**CD19**	1	PE-CY7	Biolegend	104511
**CD3**	1	PercP-Cy5.5	Biolegend	100217
**CD4**	1	PE	BD	553730
**CD45**	1 & 2	AF700	Biolegend	103128
**CD69**	1	PE-CY7	Biolegend	104511
**CD8**	1	BV605	Biolegend	100743
**F4/80**	2	PE-Vio770	Miltenyi	130-118-459
**Ly6C**	2	APC-Vio770	Miltenyi	130-111-919
**Ly6G**	2	PerCP-Vio700	Miltenyi	130-117-500
**MCHII**	2	VioBlue	Miltenyi	130-112-394
**NK1.1**	1	APC	Biolegend	108709
**Viability**	1 & 2	405/520 Fixable Dye	Miltenyi	130-110-206

Supplementary Table S3

Our cohort

a

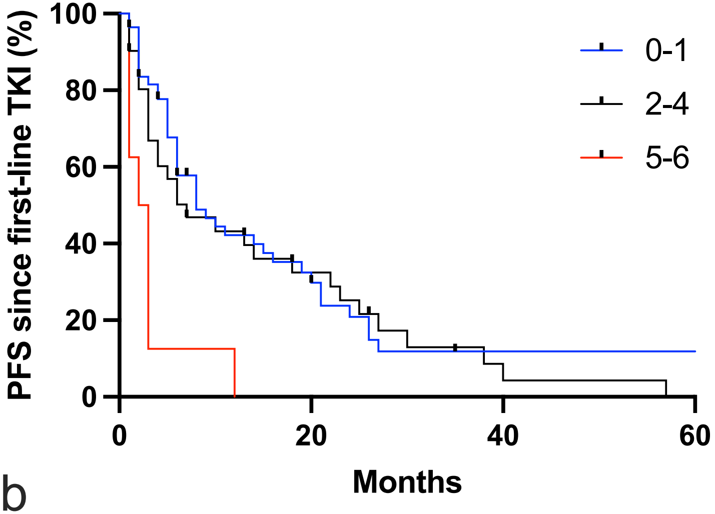

Under TKI N=98	score	PFS (months)	P value
CXCL1 + CXCL2 + CXCL3 + CXCL5 + CXCL7 + CXCL8	0	8	P=0.0097
	2-4	7	
	5-6	2.5	

b

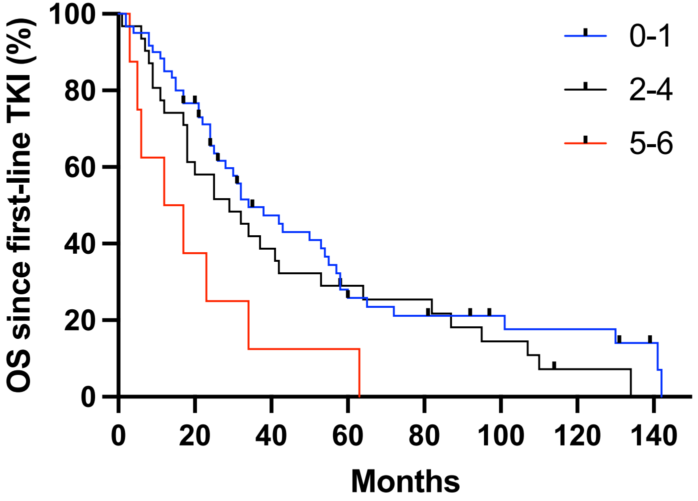

Under TKI N=98	score	OS (months)	P value
CXCL1 + CXCL2 + CXCL3 + CXCL5 + CXCL7 + CXCL8	0	34	P=0.0172
	2-4	29	
	5-6	14.5	

Supplementary Figure S1

RCT001 was synthesized by adapting the already described procedure (Grytsai et al, in revision) on a 250 mmol scale. Under these conditions, 81.45 g (85% yield) of product could be obtained, with a >99.9% HPLC purity.

^{1}H NMR (400 MHz, DMSO-d_6): δ 11.76 (s, 1H, H-10), 9.62 (s, 1H, H-12), 8.97 (s, 1H, H-6), 8.26 (dd, J = 8.9, 2.4 Hz, 1H, H-2), 7.77 (d, J = 8.8 Hz, 1H, H-3), 7.65 (s, 2H, H-15, H-19), 7.28 (t, J = 1.9 Hz, 1H, H-17).

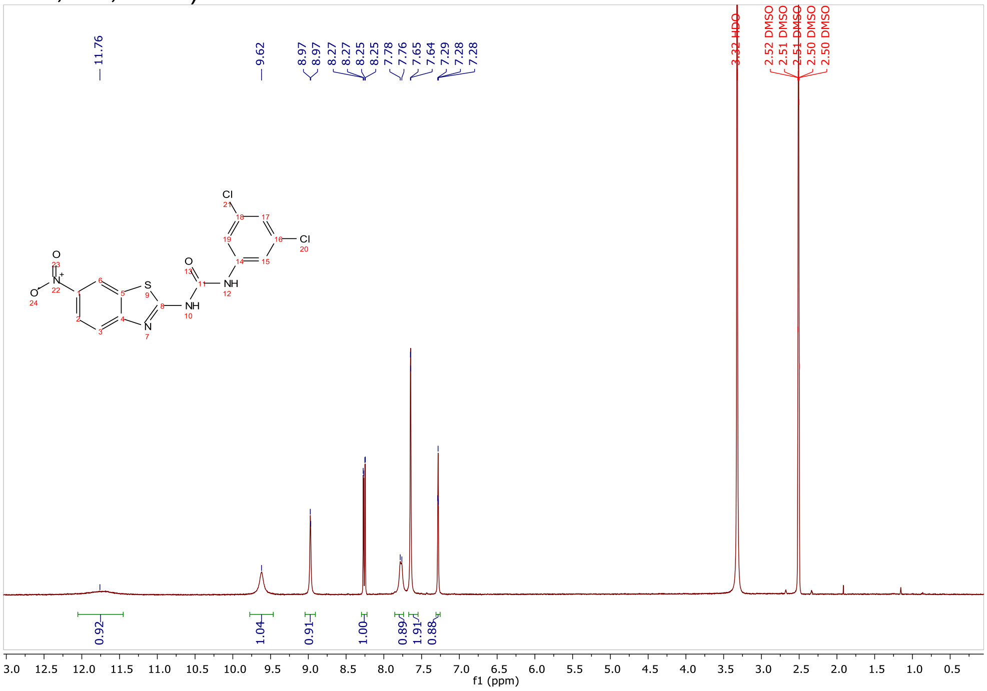

HPLC (λ$_{254}$): HPLC analysis on a JASCO PU-2089/AS4050 apparatus with a Supelco analytical column Ascentis Express C18, 10 cm × 4.6 mm, 5 µm, employing the flowing method: 50% B for 3 min, 50% B to 95% B over 2 min, 95% B for 10 min then from 95% B to 50% B over 2 min, 50% B for 3 min (total time: 20 min). Flow: 1 mL/min. Solvent A: water with 0.1% formic acid. Solvent B: acetonitrile with 0.1% formic acid.

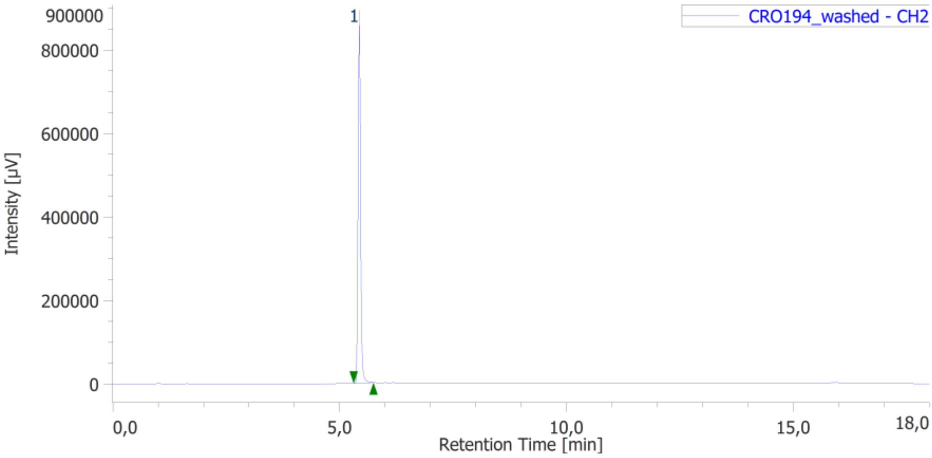

#	Peak Name	CH	tR [min]	Area [µV·sec]	Height [µV]	Area%	Height%	Quantity	NTP	Resolution	Symmetry Factor	Warning
1	Unknown	2	5.433	3293089	862920	100.000	100.000	N/A	49035	N/A	1.055	

Supplementary Figure S2

a

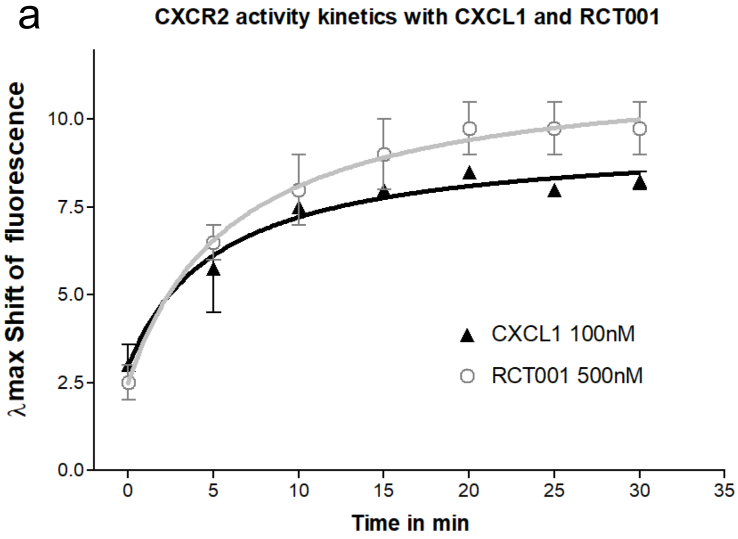

	CXCL1	RCT001
λmax (nm)	6.50	8.38
KD (min)	5.29	7.51

b

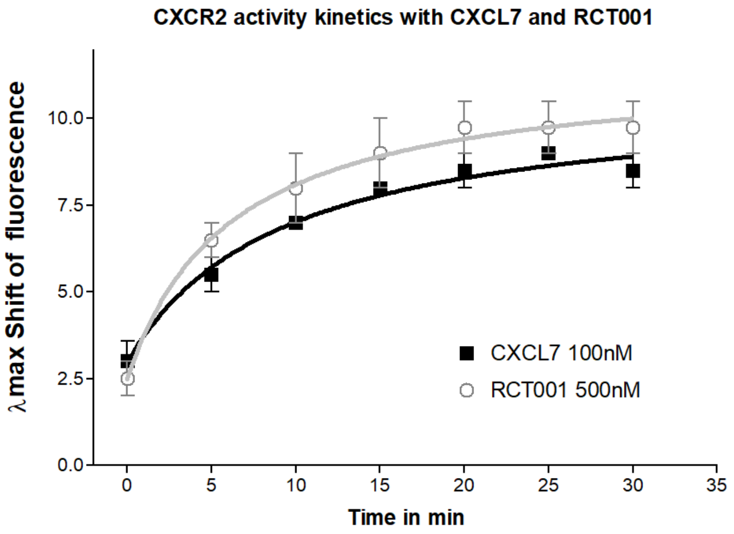

	CXCL7	RCT001
λmax (nm)	7.74	8.38
KD (min)	9.06	7.51

c

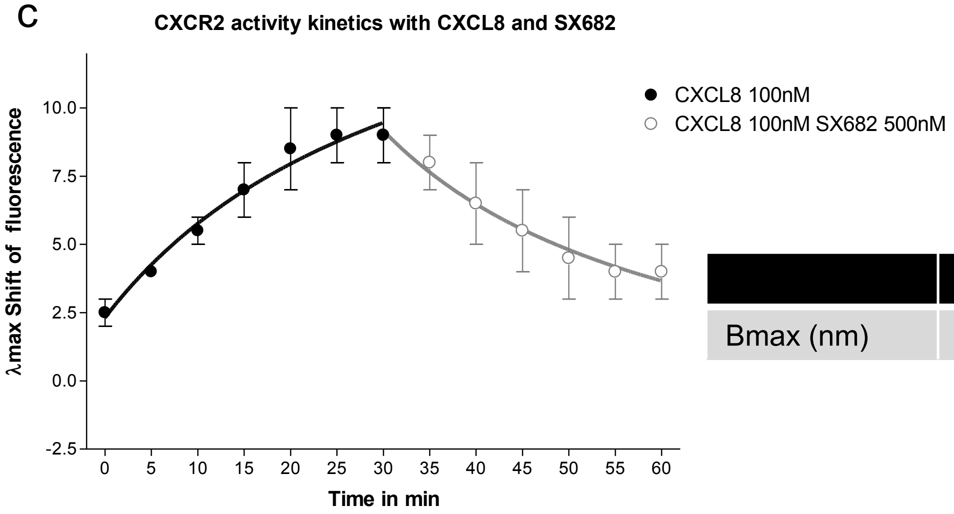

	CXCL8	SX682
Bmax (nm)	15.38	-11.49

Supplementary Figure S3

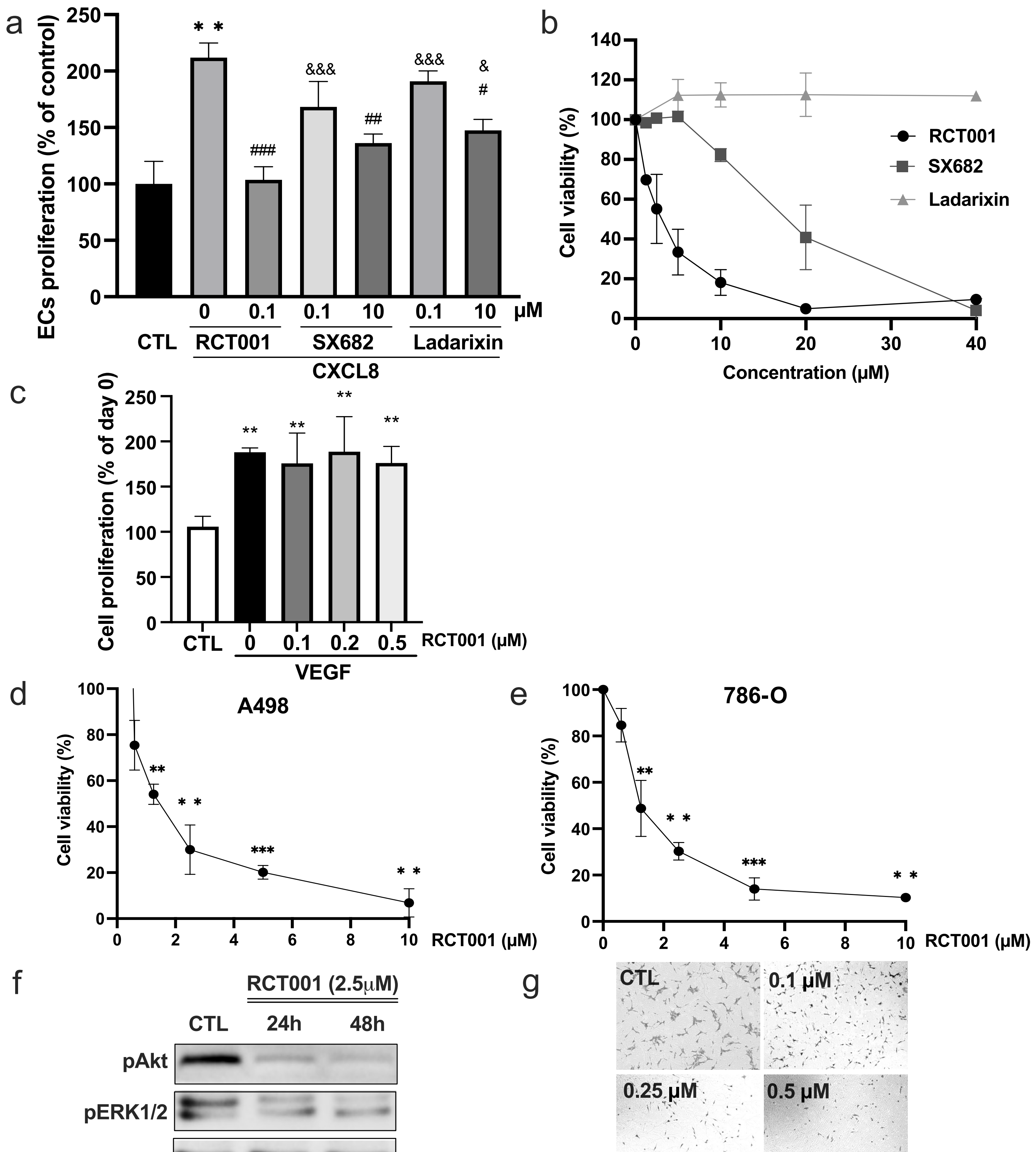

Supplementary Figure S4

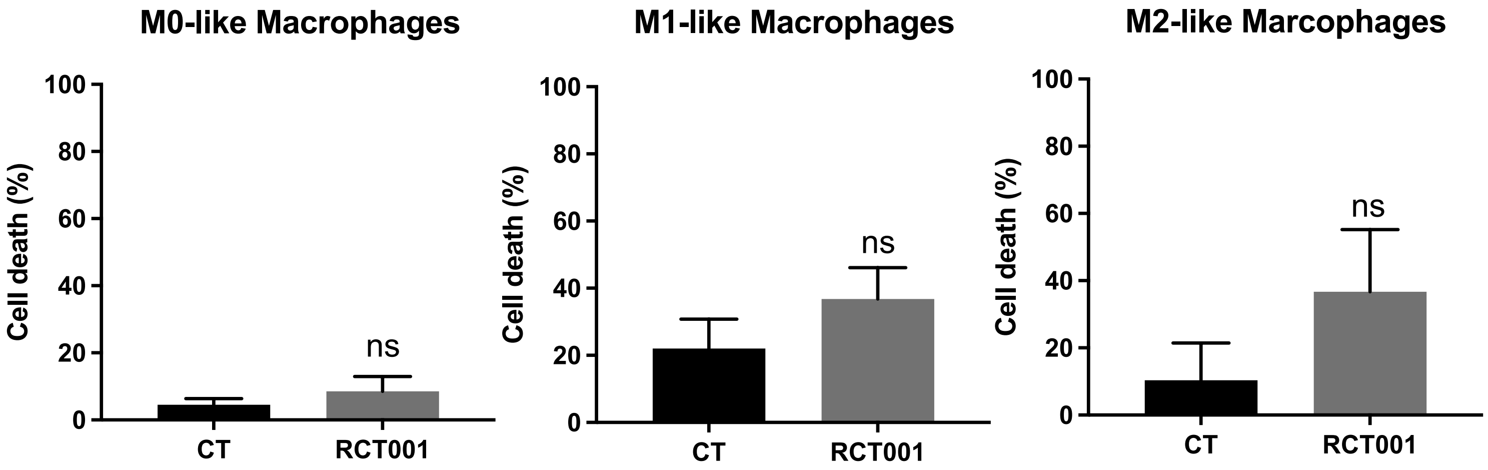

Supplementary Figure S5

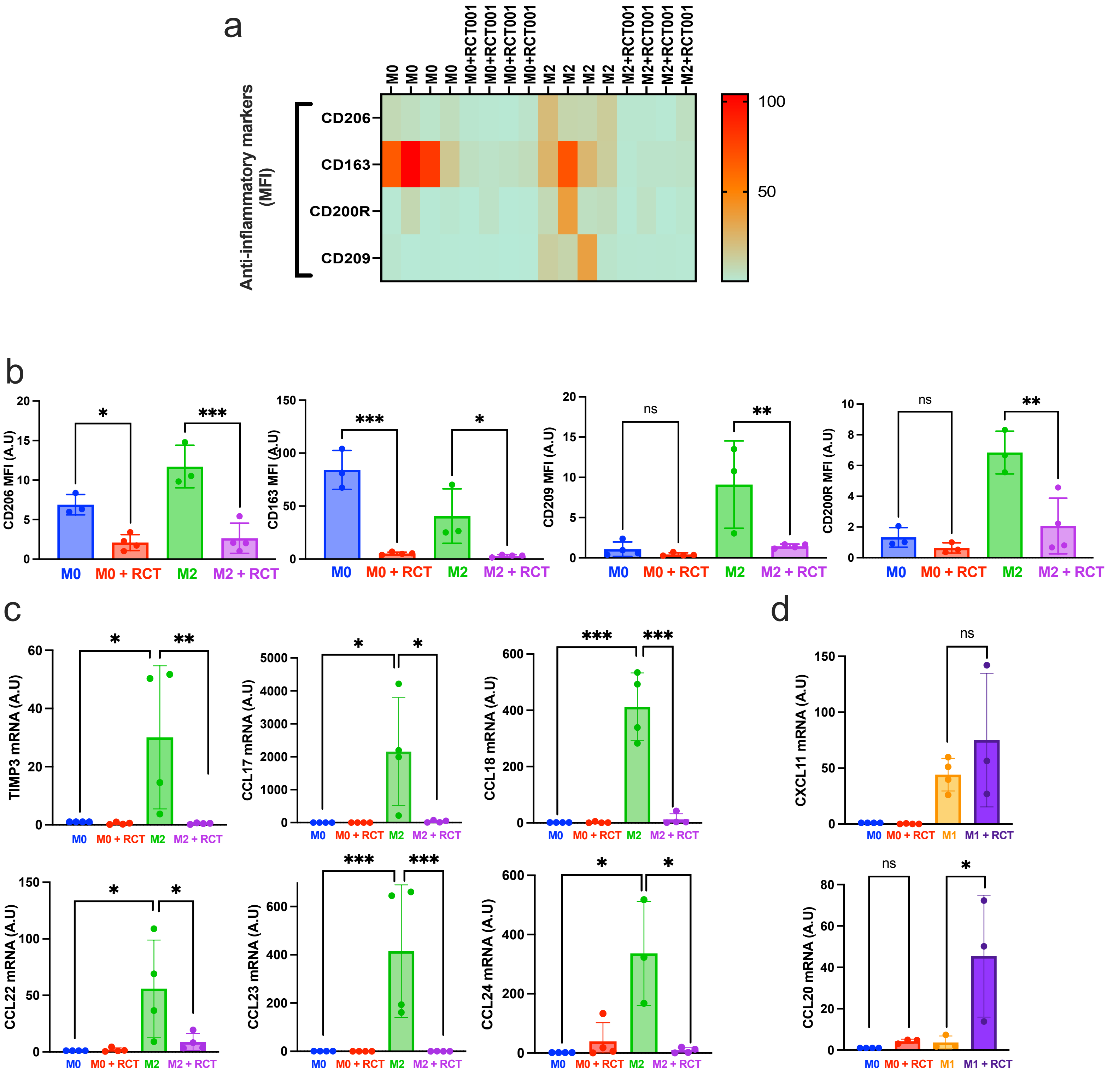

Supplementary Figure S6

M2 Macrophages

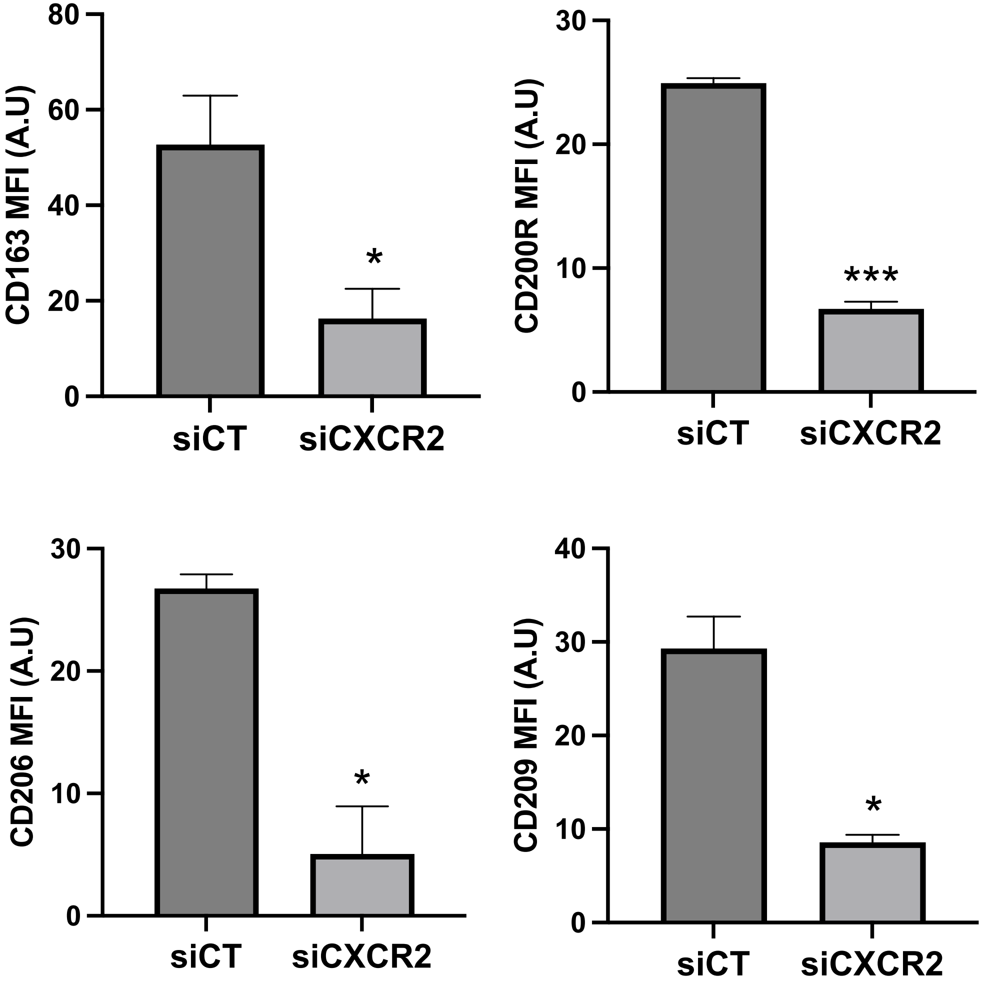

Supplementary Figure S7

a

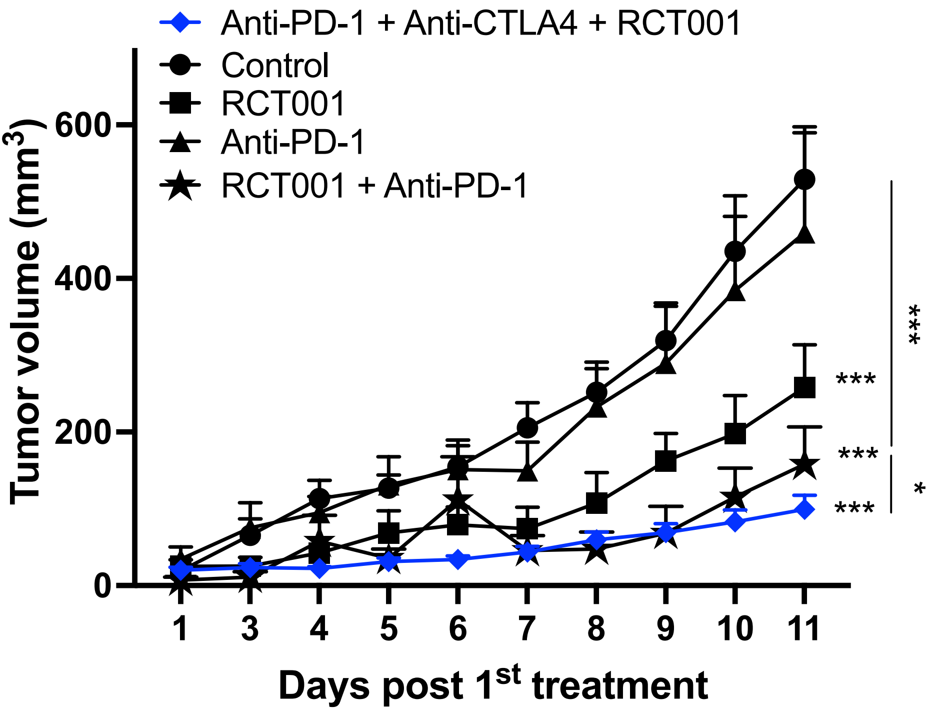

b

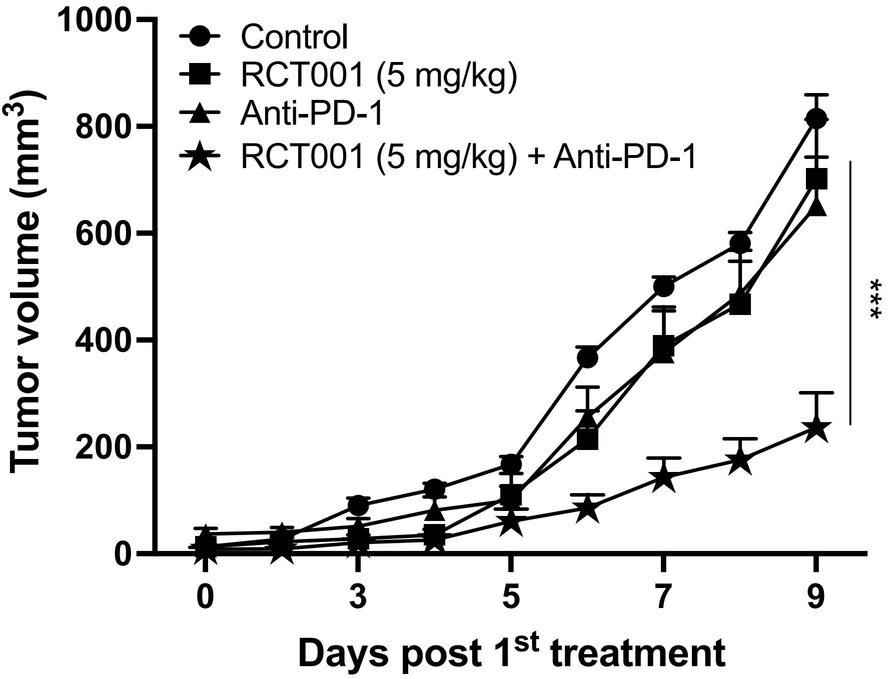

Supplementary Figure S8
