# Supplementary material for: Unveiling CXCR2 as a promising therapeutic target in renal cell carcinoma: exploring the immunotherapeutic paradigm shift through its inhibition by RCT001
Source: J Exp Clin Cancer Res. 2024 Mar 19;43:86. doi: 10.1186/s13046-024-02984-2 (PMC10949812; doi:10.1186/s13046-024-02984-2)
Supplement: Supplementary file 2 — Additional file 2. [file 13046_2024_2984_MOESM2_ESM.pdf]

Supplementary Methods

Cell lines

The following cell lines and primary cells were utilized in the study: A498 (RRID: CVCL_1056) and 786-O (RRID: CVCL_1051), RENCA (RRID: CVCL_2174), and endothelial cells (TIME, RRID: CVCL_0047), all sourced from ATCC®. The RCC cell lines were maintained in DMEM medium supplemented with 7.5% FBS. TIME cells were cultured in vascular cell basal medium (ATCC®, PCS-100-030TM) supplemented with the microvascular endothelial cell growth kit (ATCC® PCS-100-041TM). Primary RCC cells were obtained from tumor fragments, which were treated with collagenase overnight at 37°C and mechanically disaggregated using scalpels. These primary cells were then cultured in a medium designed for renal cells (PromoCell).

Evaluation of CXCR2 activity

Recombinant CXCR2 is produced and purified from HEK cells using specialized G.CLIPS proprietary mixes. The receptor is subsequently reconstituted in detergent buffers containing lipids that replicate the receptor's natural lipid composition. Following this, the receptor is labelled with a non-modifying probe. This probe enables the receptor's activation state and conformational alterations upon exposure to a ligand. Notably, the emission spectra's maximum wavelength (λmax) shifts in response to the receptor's activation or inactivation. As such, the kinetics of the receptor's activation or inactivation can be tracked by observing the λmax shift over time upon ligand introduction. This assay enables a comparative analysis of efficacy (λmax total shift) and efficiency (activation or inactivation speed) among various ligands. Consequently, it becomes possible to ascertain the direct impact of a ligand on the receptor and establish its pharmacological attributes.

Human monocyte culture and Macrophages-like differentiation

Human peripheral blood samples were collected from healthy donors with their informed consent, following the ethical principles of the Declaration of Helsinki and in accordance with recommendations set by an independent scientific review board. The blood was drawn using tubes containing ethylene diamine tetra-acetic acid (EDTA).

To isolate mononucleated cells, Ficoll Hypaque from Eurobio (CMSMSL0101) was employed. Cell enrichment was further conducted using the autoMACS® Pro Separator from Miltenyi,

France. An initial positive selection step utilizing CD14 antibodies (Miltenyi, 130-050-201) was employed to enrich monocytes.

The purified human monocytes were cultivated in RPMI 1640 medium with glutamax-I (Life Technologies, 61870044), supplemented with 10% (vol/vol) fetal bovine serum (Life Technologies). To induce macrophage differentiation, the purified monocytes were plated at a density of 0.3 × 106 per mL in RPMI 1640 medium with glutamax-I, supplemented with 10% (vol/vol) fetal bovine serum and colony-stimulating factor 1 (CSF-1). This cultivation was carried out for 5 days to yield M0-like macrophages.

To induce polarization into distinct macrophage phenotypes, M0-like macrophages were subjected to specific treatments. For M1-like polarization, lipopolysaccharide (LPS-EK Ultrapure, Invivogen, tlrl-peklps) at a concentration of 100 ng/mL and interferon gamma (Miltenyi, 130-096-484) at a concentration of 20 ng/mL were added to M0 macrophages for 48 h. M2-like polarization was achieved by adding 20 ng/mL of IL-4 (Miltenyi, 130-094-117) to M0-like macrophages for 48 h.

Following polarization, M0, M1, and M2-like macrophages were subjected to treatment with RCT001 at a concentration of 2.5 µM, applied 48 h after polarization.

Flow Cytometry Analysis

Macrophage Polarization Analysis

To assess macrophage polarization, the macrophages were detached from their culture using a solution of PBS/EDTA/bovine serum albumin (BSA). The detached cells were subsequently washed with PBS and incubated at 4°C for 10 minutes in a solution containing PBS and BSA, along with antibodies targeting specific markers:

- Anti-CD80 (Miltenyi, 130-097-204)
- Anti-CD86 (Miltenyi, 130-094-877)
- Anti-CD200R (Biolegend, 329308)
- Anti-CD206 (Miltenyi, 130-100-034)
- Anti-CD163 (Miltenyi, 130-112-130)
- Anti-CD209 (Miltenyi, 130-120-729)
- Isotype controls from Miltenyi were also used for comparison.

Macrophage Cell Death Analysis

For the analysis of macrophage cell death, the cells were washed with ice-cold phosphate-buffered saline (PBS) and then resuspended in 200 µL of PBS. Subsequently, the cells were labeled with 1 µg/mL of DAPI (4',6-diamidino-2-phenylindole) at room temperature. The

fluorescence emitted from the DAPI-labeled cells was measured using a MACSQuant® Analyzer from Miltenyi.

CXCR2 Measurement

After the desired stimulations, the cells were washed with PBS and subsequently stained with the CXCR2-PE antibody (Miltenyi) for a duration of 30 minutes at room temperature. The fluorescence resulting from this staining process was then measured using a MACSQuant® Analyzer from Miltenyi.

Tumor experiments

Study approval

All animal-related procedures conducted in this study were meticulously carried out in full compliance with the established recommendations outlined in the Guide for the Care and Use of Laboratory Animals. Additionally, all animal studies underwent rigorous evaluation and were granted prior approval by the local animal care committee, namely the Veterinary Service and the Direction of Sanitary and Social Action of Monaco.

Tumor dissociation

RENCA tumors were sectioned into small, uniform pieces measuring 2-4 mm in size. The tumor fragments were placed into a C Tube from GentleMACS (Miltenyi, 130-096-334) along with 2.5 mL of enzyme mix obtained from the Tumor Dissociation Kit, mouse (Miltenyi, 130-096-730). The tumor samples were subjected to dissociation using the GentleMACS Dissociator (Miltenyi) with the designated program 37C-m-TDK-1. The program was manually halted after 25 minutes. To remove larger debris and isolate the dissociated cells, the samples were filtered through a 70 µm MACS SmartStrainer. The filtrate was then centrifuged at 300g for 7 minutes, and the resulting pellets were resuspended in 5 mL of RPMI 1640 medium along with 427 µL of DNAse I. After incubating for 5 minutes at room temperature, the DNAse I reaction was stopped by adding 5 mL of RPMI 1640 medium supplemented with 10% fetal bovine serum (FBS). The samples were centrifuged again at 300g for 5 minutes, and the pellets were resuspended in 1 mL of RPMI 1640 medium. For the removal of red blood cells (RBCs), 9 mL of RBC Solution 1X (Miltenyi, 130-094-183) was added to each sample and incubated for 2 minutes at room temperature. The samples were then centrifuged at 300g for 10 minutes, and the pellets were resuspended in PBS along with Debris Removal Solution (Miltenyi, 130-109-398). Following this, the samples were centrifuged at 3000g for 10 minutes at 4°C. The resulting

pellets were washed in PBS and subsequently resuspended in cold PBS containing 0.5% bovine serum albumin (BSA) and 2 mM EDTA.

Flow cytometry staining step

The cells were exposed to Viability 405/520 Fixable Dye from Miltenyi and incubated for 15 minutes at room temperature in the dark. This dye enables the discrimination of live and dead cells based on their fluorescence properties. After incubation, the cells were washed and centrifuged at 1600 rpm for 2 minutes at 4°C. The cells were resuspended in 100 µL of FcBlock solution. The FcBlock solution was prepared by adding 0.5 µL of Fc Block stock per 1 million cells in 100 µL of cold PBS containing 0.5% bovine serum albumin (BSA) and 2 mM EDTA. This cell suspension was incubated for 10 minutes at 4°C. The FcBlock helps prevent non-specific binding of antibodies to Fc receptors on the cells. Following FcBlock saturation, the appropriate antibodies were added to the cells. The cells were incubated with the antibodies for 30 minutes at 4°C in the dark. These antibodies target specific markers of interest on the cells. After antibody incubation, the cells were washed and centrifuged at 1600 rpm for 2 minutes at 4°C. The cell pellets were resuspended in 100 µL of PBS containing 3% formaldehyde. This fixation step helps preserve the cell morphology and antigen integrity. The cells were incubated with the formaldehyde solution for 10 minutes at 4°C in the dark. After fixation, the cells were washed and subsequently resuspended in 100 µL of cold PBS containing 0.5% BSA and 2 mM EDTA. The stained and fixed samples were then analyzed using a Cytoflex LX cytometer. This advanced equipment allows for the measurement of fluorescence emitted by the cells, providing valuable insights into the expression of specific markers and the viability status of the cells.

Gating strategy

For FACS analysis, the following gating strategy was applied on viable cells : T cells: $CD45^+CD3^+$, CD4+: $CD45^+CD3^+CD4^+$, Activated CD4+ : $CD45^+CD3^+CD4^+CD69^+$, CD8+: $CD45^+CD3^+CD8^+$, Activated CD8+ : $CD45^+CD3^+CD8^+CD69^+$, NK: $CD45^+CD3^-CD19^-$NK1.1+, Activated NK: CD45+ $CD3^-CD19^-NK1.1^+CD107^+$, Dendritic cells (DC): $CD45^+CD3^-CD19^-NK1.1^-CD11c^+$, Activated DC: $CD45^+CD3^-CD19^-NK1.1^-CD11c^+$ MCHII high, TAMs: $CD45^+CD11b^+F4/80^+$, TAMs M1 : $CD45^+CD11b^+F4/80^+$MCHII high, TAMs M2 : $CD45^+CD11b^+F4/80^+$CD163 high, TANs: $CD45^+CD11b^+Ly6C^+Ly6G^+$.

Please refer to Supplementary Table S3 for antibody reference.

PDX Tumorspheres Establishment and Treatment

Tumorspheres derived from MEXF486 (Charles River) patient-derived xenografts (PDXs) were cultivated using a specialized process. Initially, these tumors were grown as subcutaneous

xenografts in immunodeficient mice. Upon tumor excision, single-cell suspensions were prepared. The separation of mouse cells from human tumor cells was accomplished through magnetic-activated cell sorting (MACS).

Once purified, the human tumor cells were enveloped within layers of Cypre's VersaGel®, a biocompatible hydrogel enriched with extracellular matrix components, promoting the formation of 3D tumorspheres. These tumorspheres were cultured alongside fibroblasts (HDF) and pre-activated peripheral blood mononuclear cells (PBMCs) for 7 days, after which they were subjected to treatment with RCT001 at varying doses over another 7-day period.

A separate series of experiments involved the establishment of 3D tumorspheres with HDF and pre-activated PBMCs for 7 days. Subsequently, these tumorspheres were treated with RCT001 at concentrations of 3 μM and 10 μM, alongside anti-PD-1 (pembrolizumab) at a concentration of 100 μg/mL, for additional 7 days.

The evaluation process encompassed the quantification of tumorspheres, the measurement of the total tumorspheres area, and the assessment of cell death using DRAQ7 labeling.

Peripheral blood mononuclear cells (PBMCs) for these experiments were sourced from healthy volunteers at the University Hospital in Freiburg, Germany.

Assessment of cell death using DRAQ7

After the treatment period, tumorspheres underwent an evaluation of cell death utilizing the fluorescent dye DRAQ7, provided by Biostatus Ltd, Shepshed, Loughborough, UK. This process involved incubating the tumorspheres with 300nM of DRAQ7 for a duration of 10 minutes. Following this incubation, the tumorspheres were dissociated, and the resultant samples were subjected to analysis using a FACS Calibur flow cytometer manufactured by BD Biosciences, USA. The DRAQ7 dye was excited with a wavelength of 638 nm, while the emitted fluorescence was captured through the use of a 660/16 bandpass filter.

Patients

Ethics approval

The research protocols underwent ethical approval from the ethics committee at each participating center and were conducted in accordance with the guidelines of the International Conference on Harmonization for Good Clinical Practice. Informed consent was meticulously obtained from each individual participant enrolled in the study. Written consent was secured from all patients, permitting the utilization of their tumor samples for research purposes. Importantly, the study exclusively involved adult patients, aligning with ethical considerations.

This investigation adhered to the principles outlined in the Declaration of Helsinki, underscoring its commitment to ethical research conduct.

Our cohort

Archived formalin-fixed, paraffin-embedded (FFPE) primary tumor samples and samples of resected metastases were retrieved and upon review of H&E slides, tissue blocks with the highest tumoral content were selected. Unstained slides (5 lm thickness) were produced and macrodissected to only include tumoral tissue. RNA was extracted using the Maxwell RSC RNA FFPE kit (Promega) according to the manufacturer's instructions. Subsequently, cDNA libraries were prepared using the Forward QuantSeq 30 mRNA-Seq Library Prep Kit for Illumina (Lexogen) according to the manufacturer's instructions using 5 ll of RNA and 16 PCR cycles. cDNA concentrations and fragment length were measured with the QubitTM dsDNA HS assay (Thermofisher) and Bioanalyzer HS DNA electrophoresis (Agilent). Illumina cBOT was used for clonal cluster generation and RNA sequencing was performed using the HISeq 4000 kit (Illumina) according to the manufacturer's instructions.
